# MSAT: a FAERS-informed heterogeneous graph neural network for pharmacovigilance prediction of Chinese materia medica–associated adverse drug reactions

**DOI:** 10.3389/fphar.2026.1774128

**Published:** 2026-02-26

**Authors:** Bowen Shi, Xiaojie Li, Jinghao Fang, Jisheng Chen, Jin Yang

**Affiliations:** 1 School of Medical Information and Engineering, Guangdong Pharmaceutical University, Guangzhou, Guangdong, China; 2 Guangdong Province Precise Medicine Big Data of Traditional Chinese Medicine Engineering Technology Research Center, Guangzhou, Guangdong, China; 3 Key Specialty of Clinical Pharmacy, The First Affiliated Hospital of Guangdong Pharmaceutical University, Guangdong, China

**Keywords:** adverse drug reactions, Chinese materia medica, graph neural network, pharmacovigilance, traditional Chinese medicine

## Abstract

**Background:**

Post-marketing safety surveillance of Chinese Materia Medica (CMM) is challenged by multi-component chemical heterogeneity and the limited mechanistic interpretability of signals derived solely from spontaneous reports. The FDA Adverse Event Reporting System (FAERS) provides large-scale pharmacovigilance evidence, yet it is noisy, susceptible to reporting bias, and weakly linked to underlying biological mechanisms. We aimed to develop an FAERS-informed, clinically oriented framework to predict CMM-associated adverse drug reactions (ADRs).

**Methods:**

We constructed an evidence-rich heterogeneous graph integrating CMMs, compounds, protein targets, and ADRs. To differentiate pharmacovigilance-derived statistical associations from binary molecular interactions, we augmented each CMM–ADR edge with a six-dimensional evidence feature vector (including semantic similarity, FAERS evidence as log-transformed report counts, source provenance, and topology-derived structural metrics) and used it to condition attention during message passing. We propose MSAT, a multi-scale heterogeneous graph neural network comprising: (i) an Evidence-Semantic Adaptive Gate to inject evidence-conditioned attention bias, (ii) a Hierarchical Signal Propagation layer to model cross-scale transduction from molecular mechanisms to clinical phenotypes, and (iii) a Hub-Calibrated Inference module to mitigate hub-driven bias. We evaluated MSAT using stratified 10-fold cross-validation, stress-tested robustness under increasing class imbalance up to a 1:10 positive:negative ratio, and assessed cold-start generalization. High-confidence predicted results were further examined via external database concordance and literature support.

**Results:**

In stratified 10-fold cross-validation on 27,062 curated CMM–ADR associations, MSAT achieved strong performance (AUC = 0.9792, AUPRC = 0.9766) and outperformed representative heterogeneous GNN baselines. MSAT remained robust under severe class imbalance (up to 1:10) and demonstrated favorable generalization in cold-start settings. Among the top 15 high-confidence predicted results absent from the labeled positives, 13/15 (86.7%) were supported by independent database or literature evidence. For example, MSAT prioritized a potential liver-injury signal for Aiye (*Artemisia argyi*) (predicted ADR: drug-induced liver injury, DILI), consistent with external evidence.

**Conclusion:**

By unifying FAERS pharmacovigilance evidence with multi-scale biomedical mechanisms in a heterogeneous graph learning framework, MSAT enables robust prediction and prioritization of CMM-associated ADR risks. This framework can support hypothesis generation and risk triage for post-marketing safety surveillance of complex Chinese Materia Medica products.

## 1 Introduction

Post-marketing safety surveillance of Chinese Materia Medica (CMM) remains challenging because each medicinal material contains diverse constituents and is often used in multi-component products with variable composition. Unlike single-ingredient small-molecule drugs, the multi-component and multi-target characteristics of CMM complicate both mechanistic attribution and systematic safety assessment (Ma et al., 2023; Zhang et al., 2024; Gao et al., 2019). With the expanding clinical use of CMM, particularly proprietary products and injections in routine care, reports of CMM-associated adverse drug reactions (ADRs) continue to accumulate and may include severe outcomes (Yan et al., 2022).

Spontaneous reporting systems (SRS) such as the FDA Adverse Event Reporting System (FAERS) provide indispensable real-world evidence for pharmacovigilance and early signal detection at scale (U.S. Food and Drug Administration, 2024; Harpaz et al., 2012). However, FAERS reports are inherently noisy and confounded by under-reporting, reporting bias, co-medication, and incomplete exposure information, which limits straightforward causal interpretation and weakens direct links to biological mechanisms (Harpaz et al., 2012). For complex CMM products, these limitations are further amplified by heterogeneous naming, formulation variability, and frequent concomitant use with conventional medications. A practical direction is therefore to integrate SRS evidence with mechanistic biomedical knowledge, enabling robust prioritization of safety signals and hypothesis generation for downstream assessment.

Modern computational pharmacology increasingly frames drug-safety prediction as a relational inference problem across interconnected biomedical entities. In this perspective, ADR risk emerges not only from isolated compounds but also from structured relationships among medicinal materials, chemical constituents, protein targets, and clinical phenotypes. These entities can be naturally organized as heterogeneous graphs, where link prediction supports systematic identification of unobserved associations. Graph-based learning has shown utility in modeling adverse effects in complex medication settings, including polypharmacy and multi-relational side-effect prediction (Zitnik et al., 2018). For heterogeneous biomedical graphs, relational graph neural networks (GNNs) and transformer-style architectures offer a principled mechanism to propagate information across typed nodes and edges (Schlichtkrull et al., 2018; Hu et al., 2020; Lv et al., 2021).

Nevertheless, applying heterogeneous graph learning to CMM pharmacovigilance introduces two under-addressed challenges. First, the constructed graph inevitably mixes semantically distinct edge types: molecular interactions (e.g., compound–target) are typically binary and mechanistic, whereas CMM–ADR associations derived from SRS and literature encode evidence with graded strength, provenance, and uncertainty. Many existing architectures process heterogeneous edges via uniform message passing or scalar weighting, which risks collapsing high-dimensional pharmacovigilance evidence into an underutilized auxiliary signal (Fang and Wu, 2023; Qin et al., 2021). Second, the path from molecular interactions to clinical phenotypes is intrinsically multi-scale and may require deep, long-range reasoning. Shallow propagation can suffer from over-smoothing, limiting the ability to model cross-scale signal transduction from targets to organism-level adverse outcomes (Chen et al., 2020).

Beyond algorithmic limitations, a translational gap constrains clinical utility. Model outputs are commonly expressed in regulatory terminologies such as the Medical Dictionary for Regulatory Activities (MedDRA) (Brown et al., 1999), which may not align with the diagnostic and decision frameworks used in traditional medicine practice, including Zang–Fu functional concepts (Unschuld and Tessenow, 2011; Lu et al., 2012). This mismatch can reduce the actionability of computational alerts in clinical settings where syndrome differentiation guides prescription adjustment.

To address these challenges, we propose MSAT, an FAERS-informed heterogeneous graph learning framework for pharmacovigilance prediction of CMM-associated ADRs. We first construct an evidence-rich heterogeneous graph integrating CMMs, compounds, protein targets, and ADRs, and encode pharmacovigilance evidence and provenance as edge attributes to distinguish noisy reporting signals from mechanistic interactions. MSAT introduces an Evidence-Semantic Adaptive Gate to condition attention on evidence strength, a Hierarchical Signal Propagation layer to model cross-scale transduction across CMM–compound–target–phenotype paths, and a Hub-Calibrated Inference module to mitigate degree-driven bias. We evaluate MSAT using stratified 10-fold cross-validation, stress tests under class imbalance up to 1:10 and cold-start settings, and a qualitative evidence check for representative high-confidence predicted results via database concordance and literature support. Collectively, MSAT provides a clinically aligned framework for risk prioritization and hypothesis generation in post-marketing safety surveillance of complex Chinese Materia Medica products.

## 2 Related work

Graph representation learning has been increasingly adopted for drug-safety tasks, including polypharmacy side-effect prediction and related interaction modeling (e.g., DDI prediction) (Zitnik et al., 2018; Tanvir et al., 2024b; Tanvir et al., 2021). Recent heterogeneous architectures further enrich message passing with attention mechanisms or higher-order relational designs (Tanvir et al., 2024a; Tanvir et al., 2021). While these settings differ from spontaneous-report–informed CMM pharmacovigilance, they motivate a common view of safety prediction as relational inference on heterogeneous biomedical networks. MSAT follows this perspective but is tailored to two practical issues in SRS-driven CMM–ADR modeling. First, MSAT treats CMM–ADR links as *evidence-bearing* edges and injects a multi-dimensional evidence vector into attention through ESA-Gate, rather than relying on topology or relation labels alone. Second, MSAT incorporates hub-calibrated inference to mitigate degree-driven bias that is pervasive in safety graphs (e.g., frequent ADRs and broadly connected entities), complementing heterogeneous attention with an explicit hub-bias correction mechanism. Together, these design choices address graded and uncertain pharmacovigilance evidence and reduce popularity-driven distortions in risk prioritization.

## 3 Materials and methods

**FIGURE 1:**
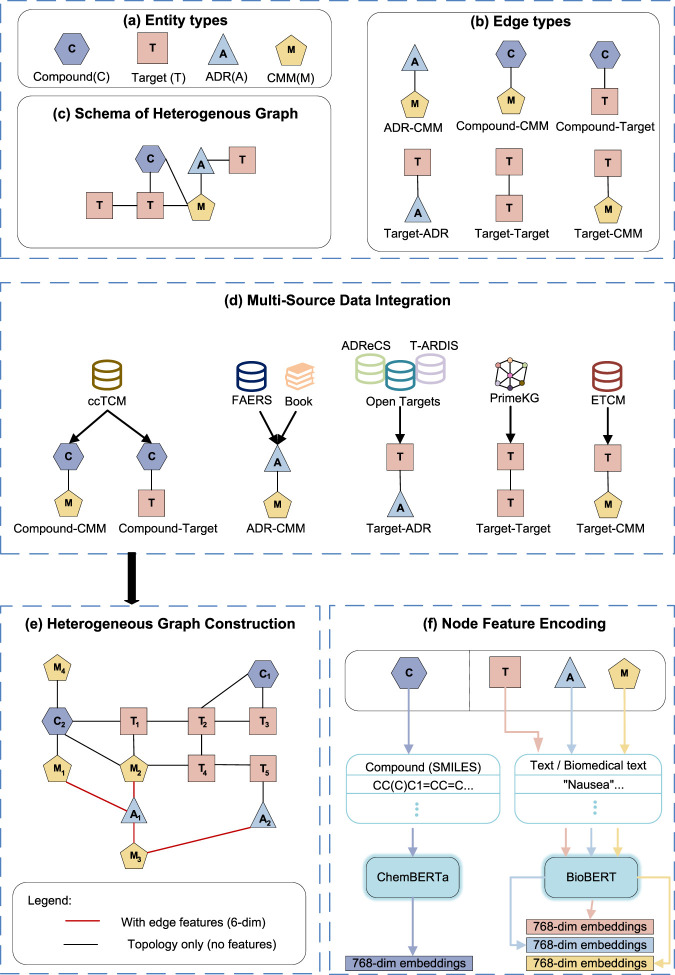
Data integration and heterogeneous graph construction. **(a–c)** Entity/edge types and the heterogeneous graph schema. **(d)** Multi-source data integration for graph assembly. **(e)** Heterogeneous graph construction with evidence-augmented edges. **(f)** Node feature encoding.

**FIGURE 2:**
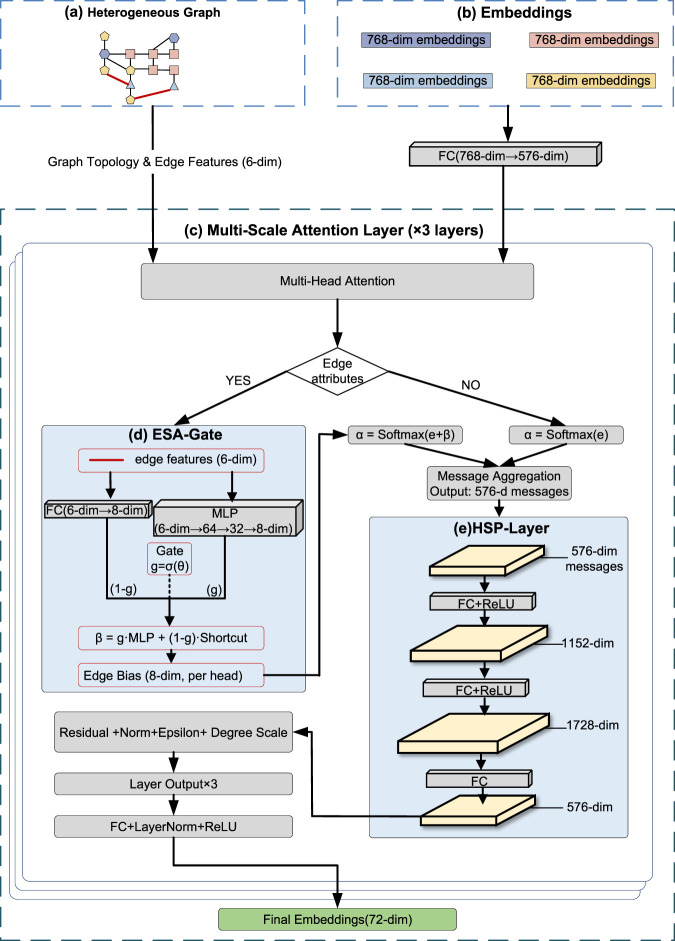
The MSAT representation learning module. **(a,b)** Inputs and embedding initialization. **(c)** Multi-Scale Attention layer ( × 3). **(d)** Evidence-Semantic Adaptive Gate (ESA-Gate). **(e)** Hierarchical Signal Propagation layer (HSP-Layer).

**FIGURE 3:**
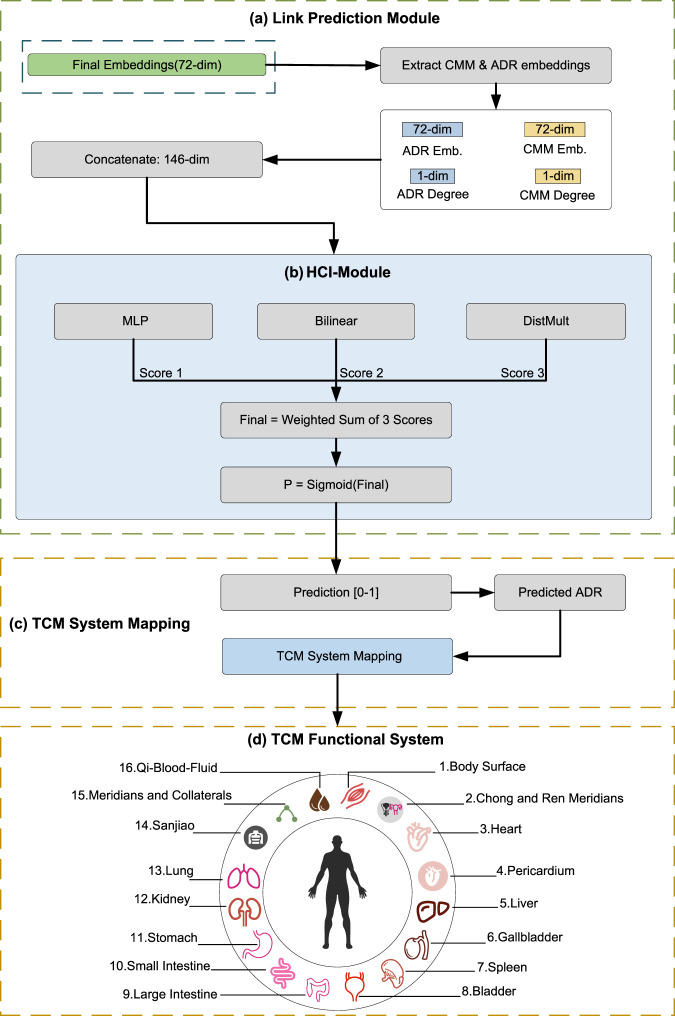
Link prediction and clinically aligned interpretation. **(a)** Link prediction for CMM–ADR associations. **(b)** Hub-Calibrated Inference module (HCI-Module). **(c,d)** Mapping predicted ADRs to TCM functional systems.

The overall methodological framework of MSAT is illustrated in Figures 1–3. It forms an end-to-end pipeline with four stages: (I) data curation and featurization, where multi-source biomedical data are integrated to construct a heterogeneous CMM–compound–target–ADR graph; (II) multi-scale representation learning with the MSAT model; (III) link prediction for CMM–ADR associations; and (IV) mapping of predicted ADRs to TCM functional systems to support clinically aligned interpretation.

### 3.1 Data collection and processing

We constructed a comprehensive dataset by integrating pharmacological data from authoritative databases (Yang et al., 2023; Chandak et al., 2023; Ochoa et al., 2023) and real-world pharmacovigilance reports (U.S. Food and Drug Administration, 2024). As summarized in Table 1, molecular associations (e.g., Compound-Target, Target-Target) were curated from structured biological repositories to form the backbone of our heterogeneous graph.

**TABLE 1:** Dataset statistics for the constructed heterogeneous graph.

Category	Type	Count	Data source(s)
Nodes	CMM	651	ccTCM, ETCM, FAERS
	Compound	1,498	ccTCM
	Protein target	21,393	ADReCS, ccTCM, PrimeKG, T-ARDIS, ETCM, Open Targets
	ADR	5,974	FAERS, Literature, ADReCS, Open Targets, T-ARDIS
	*Total nodes*	*29,516*	​
Edges	CMM-Compound	2,758	ccTCM
	Compound-Target	27,008	ccTCM
	CMM-Target	2,742	ETCM
	Target-Target	321,075	PrimeKG
	Target-ADR	30,170	ADReCS, Open Targets, T-ARDIS
	CMM–ADR	27,062	FAERS, Literature
	*Total edges*	*410,815*	​

Data source: ccTCM (Yang et al., 2023); ETCM (Xu et al., 2019); FAERS (U.S., Food and Drug Administration, 2024); Open Targets (Ochoa et al., 2023); PrimeKG (Chandak et al., 2023); ADReCS (Cai et al., 2015); T-ARDIS (Galletti et al., 2021); Literature (Lin et al., 2013).

**FIGURE 4:**
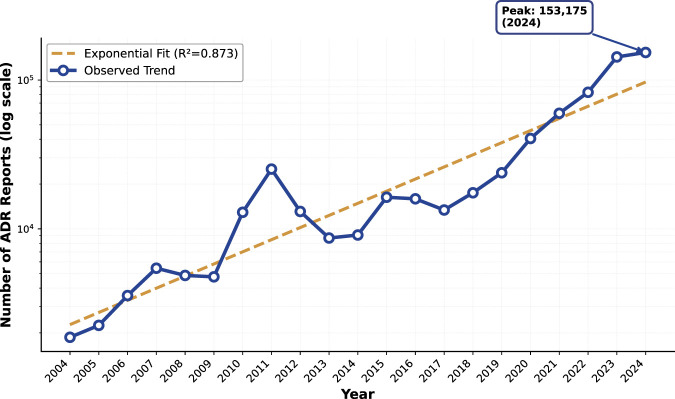
Temporal trend of CMM-related adverse event reports in FAERS (annualized, 2004–2024). The observed annualized report counts (solid line; log scale) and an exponential fit (dashed line; $R^{2}=0.873$ ) are shown. FAERS data were collected from Q1 2004 to Q1 2025; 2025 includes Q1 only and is therefore excluded from the annualized year-level plot.

To capture post-marketing safety evidence, we mined the FDA Adverse Event Reporting System (FAERS) quarterly releases from Q1 2004 to Q1 2025. We parsed the FAERS quarterly DEMO, DRUG, and REAC tables. CMM exposure mentions were identified by matching reported product names in the DRUG table against our standardized CMM lexicon (harmonized to Latin scientific names as described below), and ADR terms were taken from the REAC table as MedDRA Preferred Terms. All analyses were conducted on a locked snapshot of FAERS (through Q1 2025), which ensures reproducibility of the reported results. Figure 4 summarizes the long-term reporting trend of CMM-related adverse event records. For comparability across calendar years, the trend is presented in an annualized form; data from 2025 include Q1 only and are therefore not shown in the year-level plot.

Because spontaneous reporting data are noisy and heterogeneous, we applied a structured curation workflow before constructing CMM–ADR signals. We harmonized CMM names using TCMBank (Lv et al., 2023) and the Chinese Pharmacopoeia Commission (2025), and used standardized Latin scientific names as unique identifiers to reduce ambiguity introduced by synonyms and homonyms. Following common pharmacovigilance practice, we handled duplicate case versions by retaining the most recent version when applicable, and treated the resulting CMM–ADR associations as signals for prioritization rather than causal confirmation.

Starting from 759,122 CMM-related FAERS report–reaction records, we excluded non-medical or administrative entries by removing records mapped to 378 non-ADR MedDRA Preferred Terms (e.g., “Product issues”), which eliminated 70,120 records. We then aggregated the remaining records at the CMM–ADR level to obtain 25,734 FAERS-derived CMM–ADR associations, and supplemented them with 1,328 expert-curated associations from the literature (Lin et al., 2013). The resulting union constituted 27,062 curated positive CMM–ADR associations used as ground truth for model development; consistent with pharmacovigilance practice, these associations should be interpreted as safety signals for prioritization rather than causal confirmation.

### 3.2 Heterogeneous graph construction

We modeled the pharmacological space as a heterogeneous graph G=(V,E) comprising four node types (CMM, Compound, Target, ADR) and six relation types. Node features were initialized using domain-specific pre-trained language models: textual nodes (CMM, Target, ADR) were encoded via BioBERT (Lee et al., 2020). For compound nodes, canonical SMILES strings were retrieved from PubChem. To ensure structural consistency, we performed chemical standardization using RDKit (version 2024.03). This process involved salt removal, charge neutralization, and tautomer canonicalization before encoding sequences via ChemBERTa (Chithrananda et al., 2020). All adverse reaction terms were systematically mapped to MedDRA (Brown et al., 1999), and target protein identifiers were unified to HGNC gene nomenclature (Tweedie et al., 2021).

To differentiate pharmacovigilance-derived signals from binary mechanistic relations, we augmented each CMM–ADR edge with a six-dimensional evidence vector encoding: (1) semantic similarity between CMM and ADR descriptions (BioBERT cosine similarity); (2) FAERS evidence as log-transformed case report counts for the CMM–ADR pair; (3) a source provenance indicator (FAERS-derived vs. literature-only); and (4–6) topology-derived metrics including the CMM degree, ADR degree, and a meta-path connectivity score capturing the density of mechanistic CMM → Compound → Target → ADR paths between the pair (log-transformed and normalized). For literature-only associations without FAERS support, the FAERS count feature was set to 0 and the provenance indicator was set accordingly, while the remaining features were computed in the same manner. This evidence vector is used to condition attention during message passing, allowing evidence strength and provenance to directly modulate information propagation rather than acting as an auxiliary covariate.

#### 3.2.1 On edge provenance and leakage control

To clarify potential leakage and confounding in our heterogeneous graph, we distinguish (i) *pharmacovigilance-derived label edges* versus (ii) *independently curated mechanistic edges*. Specifically, CMM–ADR associations are mined from FAERS and supplemented by expert-curated literature and serve as the supervised link-prediction targets; these edges additionally carry evidence features (six-dimensional vectors) that encode signal strength and provenance. In contrast, mechanistic relations (CMM–Compound, Compound–Target, Target–Target, and Target–ADR) are curated from external biomedical resources and are not derived from FAERS evidence nor inferred from the CMM–ADR supervision labels. For evaluation, we use a fold-wise inductive protocol: in each cross-validation fold, all held-out positive CMM–ADR test edges are removed from the heterogeneous graph *before* training and inference, and removal is performed in both directions (CMM → ADR and ADR → CMM), with edge attributes deleted synchronously. This prevents trivial topology leakage through residual edges or evidence vectors.

### 3.3 The multi-scale attention network

The core representation learning module utilizes a stack of three Multi-Scale Attention layers. Each layer is designed for evidence-rich heterogeneous graphs and combines edge-conditioned attention with an internal non-linear transformation that supports multi-scale signal propagation from molecular interactions to clinical phenotypes.

#### 3.3.1 Evidence-semantic adaptive gate (ESA-gate)

Standard message passing paradigms typically apply a uniform aggregation function within each relation type. To resolve the semantic disparity between quantitative CMM–ADR edges and binary biological edges, we introduce ESA-Gate. For an edge feature vector $e_{ij}\in R^{6}$ , ESA-Gate generates an attention bias $\beta_{ij}$ via a learnable gating mechanism:

$$\beta_{ij}=g\cdot \text{MLP}\left(e_{ij}\right)+\left(1-g\right)\cdot \left(W_{s}e_{ij}\right)$$ (1)

Here, the gate g=σ(θ) dynamically balances two branches: a deep non-linear MLP branch, which extracts complex risk patterns from FAERS data; and a linear shortcut branch $W_{s}$ , which preserves raw statistical signals. In this way, ESA-Gate adapts its attention bias based on edge-type semantics, effectively functioning as a semantic filter for noisy pharmacovigilance signals. In practice, ESA-Gate outputs an H -dimensional vector $\beta_{ij}$ , providing one scalar bias per attention head, which is added to the corresponding head’s attention logit before softmax normalization. This bias is subsequently injected into the multi-head attention mechanism as:

$$\alpha_{ij}={\text{softmax}}_{j}\left(\frac{Q_{i}K_{j}^{T}}{\sqrt{d_{k}}}+\beta_{ij}\right)$$ (2)

where $Q_{i}$ and $K_{j}$ are the query and key vectors, $d_{k}$ is the dimension, and $\beta_{ij}$ modulates the attention logits before normalization, ensuring the model adaptively processes the diverse edge semantics described in Section 1. Unlike common edge-feature encoders that concatenate edge attributes with node representations or transform them into messages, ESA-Gate produces an additive attention bias with an explicit linear preservation path, which helps retain the scale of quantitative pharmacovigilance evidence while still allowing non-linear pattern extraction.

#### 3.3.2 Hierarchical signal propagation layer (HSP-layer)

To capture high-order hierarchical patterns from molecular binding to clinical phenotypes, we implement HSP-Layer, which mimics the hierarchical signal transduction from molecular targets to organism-level phenotypes. The aggregated message $m_{i}$ undergoes an “expand-and-compress” transformation (Hd→2Hd→3Hd→Hd) , where H is the number of attention heads and d is the per-head embedding dimension (in the default setting H=8 and d=72 , this corresponds to 576→1152→1728→576 ), inspired by biological signal transduction motifs:

$$\text{HSP}\left(m_{i}\right)=W_{3}\cdot \sigma \left(W_{2}\cdot \sigma \left(W_{1}m_{i}\right)\right)$$ (3)

This structure projects features into a high-dimensional manifold to disentangle the non-linear dependencies inherent in multi-component CMM formulations before projecting them back for residual connection. By increasing capacity within a single layer and keeping a residual pathway, HSP-Layer reduces the need for deeper stacking, which is often unstable in biomedical graphs due to over-smoothing and noisy long-range propagation.

### 3.4 Link prediction and clinical alignment

The final stage of MSAT is designed to bridge the translational gap between computational outputs and clinical practice.

#### 3.4.1 Hub-calibrated inference module (HCI-module)

To capture association patterns that include both symmetric functional similarity and asymmetric directional mechanisms, we employ a multi-perspective prediction module. The module computes a probability score via the weighted fusion of three functions:

$$\begin{array}{cc} s_{\text{final}}= & \;w_{1}\cdot \text{MLP}\left(\left[z_{\text{CMM}}\|z_{\text{ADR}}\|\deg_{\text{CMM}}\|\deg_{\text{ADR}}\right]\right)\\ & +w_{2}\cdot \left(z_{\text{CMM}}^{T}W_{B}z_{\text{ADR}}\right)\\ & +w_{3}\cdot \left(z_{\text{CMM}}⊙z_{\text{ADR}}\right) \end{array}$$ (4)

Here, the MLP scorer incorporates node degrees (deg) to account for hub effects; the bilinear term $\left(W_{B}\right)$ captures directional (asymmetric) association patterns; and DistMult (⊙) models symmetric similarity. Placing degree information in the MLP scorer and learning fusion weights allows HCI-Module to calibrate hub effects at the scoring stage, complementing representation learning without forcing degree heuristics into message passing.

The complete algorithmic workflow of MSAT is summarized in Algorithm 1.

Algorithm 1 **Proposed MSAT framework.**

**Require:** Heterogeneous graph G ; training set $\left(S_{\text{tr}},y_{\text{tr}}\right)$

**Ensure:** Clinically aligned predictions P

 1: **procedure** BuildMSAT( G )
 2:  Encode nodes via BioBERT (CMM, Target, ADR)/ChemBERTa (Compound)
 3:  Project all node features to hidden dimension $d_{h}$

 4:  **for** ℓ=1 to L **do**
 5: $\beta^{\left(\ell \right)}\leftarrow \text{ESA}-\text{Gate}\left(e_{ij}\right)$ ⊳ Equation 1
 6: $h^{\left(\ell \right)}\leftarrow \text{Attention}\left(h^{\left(\ell -1\right)},\beta^{\left(\ell \right)}\right)$ ⊳ Equation 2
 7: $h^{\left(\ell \right)}\leftarrow \text{LayerNorm}\left(\text{HSP}\left(h^{\left(\ell \right)}\right)+h^{\left(\ell -1\right)}\right)$ ⊳ Equation 3
 8:  **end for**
 9:  **return** Embeddings $z_{c},z_{a}$ and HCI predictor
 10: **end procedure**
 11: **procedure** Train(MSAT, $S_{\text{tr}}$ , $y_{\text{tr}}$ )
 12:  Optimize via AdamW with BCE loss; early stop on validation AUC
 13: **end procedure**
 14: **procedure** Predict(MSAT, $S_{\text{val}}$ )
 15:  **for** $\left(c,a\right)\in S_{\text{val}}$ **do**
 16: $s\leftarrow \text{HCI}\left(z_{c},z_{a},\deg\left(c\right),\deg\left(a\right)\right)$ ; p(c,a)←σ(s) ⊳ Equation 4
 17:   Map ADR a to TCM functional system(s)
 18:  **end for**
 19:  **return** P with probabilities and TCM mappings
 20: **end procedure**

#### 3.4.2 Ontology alignment with traditional medical systems

To translate computational predictions into clinically interpretable phenotypes, we implemented a rule-based ontology alignment layer. This layer maps the standardized MedDRA terminology to the Zang–Fu functional ontology based on the MedDRA hierarchy. First, each MedDRA Preferred Term (PT) is mapped to its corresponding System Organ Class (SOC). Second, expert-defined mapping rules take both the PT and SOC as inputs to assign one or more TCM functional systems. This dual-input logic is critical: for example, within the Gastrointestinal disorders SOC, “Jaundice” is mapped to the Liver system, whereas “Nausea” is mapped to the Spleen and Stomach systems. Mapping rules were drafted and iteratively refined through expert consensus review by clinicians trained in traditional medicine, and all assignments were manually checked for internal consistency. The final schema comprises 16 functional systems (five Zang organs, six Fu organs, and the Body Surface, Chong-Ren meridians, Meridians and Collaterals, and Qi-Blood-Fluid systems), as summarized in Figures 3c,d. This mapping enables MSAT to provide evidence-based risk assessments expressed directly in TCM diagnostic terms.

### 3.5 Experimental setup

#### 3.5.1 Data splitting and cross-validation strategy

We employed stratified 10-fold cross-validation on the 27,062 CMM–ADR associations to ensure robust performance evaluation and reduce the risk of overfitting. The dataset was randomly partitioned into ten folds with an approximately equal distribution of positive samples across folds. In each iteration, one fold served as the test set, and the remaining nine folds formed the development data. Within each development set (90% of the full dataset), we randomly allocated 90% of the samples for model training and 10% for validation, yielding an overall approximate 81:9:10 split across train/validation/test sets. The validation set was used to monitor model performance during training, and the checkpoint with the highest validation AUC was saved for final evaluation on the corresponding test fold.

For each fold, all positive CMM–ADR edges in the held-out test split were removed from the heterogeneous graph before model training to prevent information leakage through graph topology during test evaluation; removal was performed in both directions and with edge attributes deleted synchronously.

##### 3.5.1.1 Negative sampling for sampled binary evaluation

For the main experiments, we constructed a sampled binary dataset with a fixed positive-to-negative ratio of 1:1 using *type-constrained* negative sampling: for each positive CMM–ADR pair (h,a) , we fixed the CMM h and sampled one alternative ADR $a^{\prime }$ uniformly from the full ADR set to form a negative pair $\left(h,a^{\prime }\right)$ . To reduce false negatives, any sampled $\left(h,a^{\prime }\right)$ that appeared in the union of known positives within the current fold (train/validation/test positives) was discarded, and duplicate negatives for the same positive were removed. To ensure fairness and reproducibility, negative samples were generated once per fold and per split (train/val/test) using a fixed random seed, saved, and reused across all models. For the end-to-end 1:10 imbalanced setting (Table 4), we applied the same type-constrained sampling and filtering rules, but sampled 10 negatives per positive for train, validation, and test splits. Importantly, sampled negatives correspond to unobserved CMM–ADR pairs rather than confirmed non-associations; this evaluation setting reflects standard practice in link prediction under incomplete pharmacovigilance knowledge. As sensitivity analyses, we additionally evaluated MSAT under stricter FAERS-only supervision with increasing minimum report-count thresholds and under mechanism-aware hard-negative sampling. Performance remained stable across these stress-test settings (Supplementary Tables S2–S3).

##### 3.5.1.2 Evaluation settings and why 1:1 is reported

We evaluated MSAT under three complementary settings.

- Balanced evaluation ( 1:1 negative sampling): used to provide a controlled and comparable benchmark for model selection and ablation, mitigating metric inflation caused by extreme class skew.
- Imbalance stress tests: we progressively increased the negative-to-positive ratio up to 1:10 at evaluation time to reflect real-world sparsity and assess robustness.
- End-to-end imbalanced training and testing (1:10) **:** we further trained and evaluated models under 1:10 sampling throughout the pipeline, representing a more deployment-aligned scenario.

Therefore, the 1:1 setting is reported as a standardized reference point, while the 1:10 settings provide the deployment-oriented robustness evidence.

#### 3.5.2 Model training and hyperparameter configuration

All models (MSAT and nine baseline methods) were trained with consistent configurations to ensure fair comparison. MSAT employed the following hyperparameters: per-head embedding dimension d=72 with eight attention heads (total multi-head message size 8d=576 ), three Multi-Scale Attention layers (L=3) , dropout rate 0.18, and edge feature dimension six. The model was optimized using the AdamW optimizer with a learning rate of $4\times 10^{-4}$ , weight decay $1\times 10^{-5}$ , and batch size 512. Training proceeded for a maximum of 1,000 epochs per fold with early stopping patience of 100 epochs (terminating if validation AUC did not improve for 100 consecutive epochs). Gradient clipping with a maximum norm of 1.0 was applied to prevent gradient explosion. A ReduceLROnPlateau scheduler with factor 0.6 and patience 15 was used to decrease the learning rate when validation AUC plateaued.

Baseline methods were configured with comparable model capacities where applicable. Graph neural network baselines (GCN (Kipf and Welling, 2017), GAT (Veličković et al., 2018), R-GCN (Schlichtkrull et al., 2018), HGT (Hu et al., 2020), Simple-HGN (Lv et al., 2021), and HetGNN (Zhang et al., 2019)) used hidden dimensions and layer numbers adjusted to achieve similar parameter counts as MSAT. Traditional machine learning baselines (logistic regression (LaValley, 2008), random forest (Rigatti, 2017), XGBoost (Chen and Guestrin, 2016)) were trained on flattened node features and CMM–ADR edge features without using graph structure. All models used identical train/validation/test splits and negative sampling strategies to eliminate confounding factors. Full hyperparameter configurations for all baseline models are detailed in Additional file 1.

#### 3.5.3 Performance evaluation metrics and experimental protocols

Model performance was assessed using six complementary metrics. Let TP and TN denote the numbers of correctly predicted CMM–ADR associations and non-associations, and FP and FN the numbers of false positives and false negatives, respectively. Precision and recall are defined as follows (Equation 5):

$$\text{Precision}=\frac{TP}{TP+FP},\;\text{Recall}=\frac{TP}{TP+FN}.$$ (5)

The F1-score is the harmonic mean of precision and recall (Equation 6):

$$\text{F}1=2\cdot \frac{\text{Precision}\cdot \text{Recall}}{\text{Precision}+\text{Recall}}.$$ (6)

The Matthews correlation coefficient (MCC) provides a balanced summary of binary classification quality and takes values in [−1,1] (Equation 7):

$$\text{MCC}=\frac{TP\times TN-FP\times FN}{\sqrt{\left(TP+FP\right)\left(TP+FN\right)\left(TN+FP\right)\left(TN+FN\right)}}.$$ (7)

AUC (AUROC; area under the receiver operating characteristic curve) evaluates discrimination across all decision thresholds, while AUPRC (area under the precision–recall curve) is particularly informative under class imbalance by emphasizing the high-precision regime. Throughout the manuscript, we use AUC to denote AUROC.

##### 3.5.3.1 Thresholding protocol

We report threshold-independent metrics (AUC and AUPRC). For threshold-dependent metrics (Precision, Recall, F1-score, and MCC), we used two evaluation protocols. (i) Main experiments: predicted association probabilities were binarized using a fixed threshold of 0.5 for all models and all folds. (ii) Imbalanced and end-to-end 1:10 evaluations: the decision threshold $\tau^{*}$ was selected solely on the validation set by maximizing the F1-score (ties broken by choosing the smaller threshold), and then applied unchanged to the matched test set. To support threshold selection under realistic imbalance, we additionally provide precision–recall curves for the 1:10 proxy setting and mark the operating points corresponding to τ=0.5 and $\tau^{*}$ (Supplementary Figure S3). The PR curves show the expected trade-off between alert burden (precision) and sensitivity (recall). Reporting both a fixed threshold and a validation-selected operating point provides a practical range for downstream pharmacovigilance triage without test-set tuning (Supplementary Figure S3).

All results are reported as the mean ± standard deviation across the ten cross-validation folds.

For statistical comparison of AUROC, we performed paired tests on sample-level out-of-fold prediction scores by concatenating the held-out predictions from all 10 folds (pooled out-of-fold evaluation). p -values in Tables 2 and 3 are computed by comparing each baseline against MSAT under identical splits, and are reported in a thresholded form (e.g., $p<10^{-4}$ ) for compact presentation. Unless otherwise stated, statistical significance annotations (e.g., in Figure 3) were computed using two-sided paired t -tests on fold-wise metric values (paired by identical data splits).

**TABLE 2:** Performance of MSAT and baseline models for CMM–ADR prediction.

Model	Precision	Recall	F1-score	AUC	AUPRC	MCC	p (AUC)
LR	0.833 ± 0.009	0.873 ± 0.007	0.852 ± 0.006	0.931 ± 0.004	0.923 ± 0.006	0.699 ± 0.010	$<10^{-10}$
RF	0.841 ± 0.005	0.869 ± 0.007	0.854 ± 0.004	0.920 ± 0.003	0.898 ± 0.007	0.704 ± 0.006	$<10^{-10}$
XGBoost	0.842 ± 0.007	0.871 ± 0.006	0.856 ± 0.004	0.933 ± 0.002	0.927 ± 0.004	0.708 ± 0.007	$<10^{-10}$
GCN	0.930 ± 0.006	0.913 ± 0.006	0.921 ± 0.004	0.968 ± 0.003	0.972 ± 0.003	0.844 ± 0.007	$<10^{-5}$
GAT	0.925 ± 0.009	0.884 ± 0.010	0.904 ± 0.005	0.969 ± 0.003	0.965 ± 0.004	0.813 ± 0.008	$<10^{-7}$
R-GCN	**0.945** ± **0.006**	0.771 ± 0.012	0.849 ± 0.009	0.944 ± 0.004	0.945 ± 0.003	0.739 ± 0.012	$<10^{-10}$
HGT	0.932 ± 0.006	0.857 ± 0.008	0.893 ± 0.005	0.970 ± 0.002	0.968 ± 0.003	0.797 ± 0.008	$<10^{-8}$
HetGNN	0.870 ± 0.011	0.924 ± 0.013	0.896 ± 0.004	0.949 ± 0.004	0.948 ± 0.004	0.788 ± 0.009	$<10^{-8}$
Simple-HGN	0.937 ± 0.007	0.858 ± 0.010	0.896 ± 0.006	0.972 ± 0.002	0.971 ± 0.002	0.803 ± 0.010	$<10^{-7}$
MSAT	0.927 ± 0.005	**0.934** ± **0.005**	**0.930** ± **0.003**	**0.979** ± **0.001**	**0.977** ± **0.002**	**0.860** ± **0.005**	–

Values are reported as mean ± standard deviation over stratified 10-fold cross-validation. p -values indicate the significance of the performance difference between MSAT and each baseline (paired t -test on AUC). Abbreviations: LR, logistic regression; RF, random forest.

p(AUC) is computed from paired tests on pooled out-of-fold sample-level predictions (baseline vs. MSAT) and displayed in a thresholded form for compactness; “–” indicates the reference model.

Bold values indicate the best performance for each evaluation metric.

**TABLE 3:** Ablation study of MSAT components for CMM–ADR prediction.

Variant	Precision	Recall	F1-score	AUC	AUPRC	MCC	p (AUC)
Full	0.927 ± 0.005	**0.934** ± **0.005**	**0.930** ± **0.003**	**0.979** ± **0.001**	**0.977** ± **0.002**	**0.860** ± **0.005**	–
w/o HCI-Module	0.929 ± 0.005	0.895 ± 0.020	0.912 ± 0.010	0.975 ± 0.002	0.972 ± 0.002	0.827 ± 0.017	$<10^{-4}$
w/o HSP-Layer	**0.939** ± **0.007**	0.838 ± 0.009	0.885 ± 0.004	0.965 ± 0.002	0.963 ± 0.003	0.788 ± 0.006	$<10^{-7}$
w/o ESA-Gate	0.936 ± 0.006	0.849 ± 0.014	0.890 ± 0.007	0.968 ± 0.003	0.966 ± 0.003	0.794 ± 0.010	$<10^{-6}$
Only ESA-Gate	0.922 ± 0.006	0.909 ± 0.010	0.915 ± 0.005	0.973 ± 0.002	0.970 ± 0.002	0.832 ± 0.009	$<10^{-7}$
Only HSP-Layer	0.918 ± 0.011	0.908 ± 0.015	0.913 ± 0.006	0.971 ± 0.002	0.968 ± 0.003	0.827 ± 0.011	$<10^{-7}$
Only HCI-Module	0.934 ± 0.005	0.834 ± 0.010	0.881 ± 0.007	0.961 ± 0.003	0.961 ± 0.004	0.780 ± 0.012	$<10^{-7}$

Values are reported as mean ± standard deviation over stratified 10-fold cross-validation.

p(AUC) is computed from paired tests on pooled out-of-fold sample-level predictions (baseline vs. MSAT) and displayed in a thresholded form for compactness; “–” indicates the reference model.

Bold values indicate the best performance for each evaluation metric.

We conducted a series of experiments to comprehensively evaluate MSAT, including (i) overall comparison with nine baseline methods under the standard balanced setting, (ii) robustness under varying class-imbalance ratios, (iii) cold-start generalization across data sources, (iv) degree-stratified bias analysis, and (v) parameter sensitivity and ablation studies. The detailed protocols for these experiments are provided in Section 3.5.4.

#### 3.5.4 Robustness, generalization, and fairness protocols

Beyond the primary cross-validation experiments, we designed four additional protocols to evaluate the robustness, generalization, and fairness of MSAT in clinically realistic settings.

- Class-imbalance stress testing (clinical reality simulation). In real-world pharmacovigilance, true CMM-induced adverse reactions are rare relative to non-events. To mimic this sparsity, we constructed test sets with increasing negative-to-positive ratios of 1:1, 1:2, 1:5, and 1:10. For each cross-validation fold, the training data remained balanced at a 1:1 ratio as described in Section 3.5.1. To create an imbalanced test set for a given ratio, we retained all positive CMM–ADR pairs from the held-out fold and sampled additional negatives using the same type-constrained strategy (fixing the CMM and replacing the ADR), from the pool of unseen non-edges, avoiding any pair used in training or validation. For each model, ratio, and fold, we selected a decision threshold on the corresponding validation set by maximizing the F1-score, and then applied this ratio-specific threshold to the test set to evaluate precision, recall, F1, and MCC. AUC and AUPRC were calculated to characterize ranking robustness under varying imbalance levels. For each fold and each ratio, the sampled test negatives were generated once with a fixed seed and reused across all models.
- Cold-start generalization across data sources (external validity). To assess whether MSAT can generalize across heterogeneous data sources, we designed a source-transfer experiment. The model was trained exclusively on noisy statistical associations between CMMs and ADRs mined from the FAERS pharmacovigilance database and evaluated on an expert-curated set of high-quality CMM–ADR associations extracted from the literature, which were held out from training. This setting tests whether patterns learned from large-scale statistical signals can transfer to mechanistically plausible, expert-verified cases.
- Degree-stratified bias analysis (hubness bias). Biomedical networks often exhibit highly skewed degree distributions, which can cause models to over-rely on popular, high-degree nodes. To examine whether MSAT disproportionately favors well-studied CMMs, we stratified test CMM nodes into three groups based on their degree in the CMM-centered subgraph: *Head* (top 20% by degree), *Medium* (middle 60%), and *Tail* (bottom 20%). We then reported all evaluation metrics separately for each group and compared performance gaps between head and tail CMMs to quantify degree-related bias.
- Parameter sensitivity and ablation studies (internal validity). To disentangle architectural contributions from hyperparameter effects, we conducted sensitivity analysis over two key structural parameters: the per-head embedding dimension ( d∈{32,64,96,128} ; with eight heads, the total multi-head message size is 8d ) and the number of MSAT layers (L∈{1,2,3,4}) . For each configuration, we retrained MSAT under the same 10-fold protocol and reported all metrics. In addition, we performed stepwise ablation of MSAT’s main components by removing the Evidence-Semantic Adaptive Gate (ESA-Gate), the Hierarchical Signal Propagation Layer (HSP-Layer), and the Hub-Calibrated Inference Module (HCI-Module), respectively. This allowed us to quantify the contribution of each module to overall performance and to verify that the observed improvements are attributable to the proposed architecture rather than incidental hyperparameter tuning. We further conducted data-source-level ablations that selectively remove (i) semantic node features (compound/target/ADR/CMM embeddings) or (ii) key mechanistic relations (e.g., target–target PPI edges and target–ADR edges) to quantify the marginal contribution of each heterogeneous information source (Supplementary Tables S4). Among the examined sources, ablating phenotype (ADR) information yields the largest degradation, followed by target and compound information, whereas removing pathway/PPI edges has a comparatively smaller but non-negligible effect, supporting that MSAT benefits from both semantic embeddings and mechanistic connectivity.

#### 3.5.5 Implementation details

Experiments were conducted on a computing system equipped with an NVIDIA L20 GPU (46 GB memory). The model was implemented in Python 3.10 using PyTorch 2.4 with CUDA 12.1 for deep learning operations and PyTorch Geometric for heterogeneous graph neural network layers. Pre-trained BioBERT and ChemBERTa models were obtained from Hugging Face Transformers library. Statistical analyses and visualization were performed using NumPy, SciPy, Pandas, and Matplotlib. All experiments used random seed 42 for reproducibility. The complete source code is publicly available at https://github.com/BowenShiGDPU/MSAT.

#### 3.5.6 Validation strategy for predicted results

To evaluate the plausibility of MSAT’s ranked predicted results while addressing selection bias, we used a two-part validation design. First, for qualitative interpretability, we report the Top 15 highest-confidence predicted CMM–ADR associations that were not included among labeled positives in the curated supervision. Second, to assess reliability beyond the extreme top of the ranking, we *uniformly randomly sampled* two additional sets of predictions from lower-confidence strata: 15 pairs from ranks 21–100 and 15 pairs from ranks 101–200. We intentionally describe these items as *predicted results* rather than “new discoveries”; the supervision signal (FAERS-derived labels supplemented by curated literature) is incomplete, and under-reporting as well as conservative term normalization/filtering can leave some clinically recognized effects unlabeled. All predictions were evaluated using the same two-channel validation protocol. (i) Database verification: we queried the Traditional Chinese Medicine and Active Ingredients Database (Shanghai Institute of Organic Chemistry, Chinese Academy of Sciences, 2024) and considered a prediction supported if the database listed an adverse phenotype that directly matched (or was a close synonym of) the predicted MedDRA Preferred Term. (ii) Literature/mechanistic support: we performed targeted literature review to identify either direct evidence linking the CMM to the ADR, or indirect mechanistic evidence linking known constituents/targets to the ADR phenotype. For a conservative screening-style definition aligned with pharmacovigilance triage, we count a prediction as *validated* if either channel provides support (logical OR). Full lists for the randomly sampled strata are provided in Supplementary Tables S5–S8.

## 4 Results

### 4.1 Overall predictive performance

We first compared MSAT with nine baseline methods using stratified 10-fold cross-validation on the CMM–ADR prediction task. As shown in Table 2, MSAT achieved the best performance on every metric against all baseline models. It obtained the highest AUC, AUPRC, and Matthews correlation coefficient, indicating strong discriminative ability in ranking CMM–ADR associations and a favorable balance between sensitivity and specificity. Compared with the strongest heterogeneous baseline (Simple-HGN) and the strongest homogeneous baseline (GCN), MSAT achieved higher recall and MCC, and the improvements in AUC were statistically significant across all baselines ( $p<10^{-5}$ ; Table 2).

In comparison to other models, MSAT showed different strengths. Against heterogeneous GNNs, MSAT achieved similar precision but substantially higher recall, indicating that standard heterogeneous models tend to under-predict true ADRs. Compared with homogeneous GNNs and traditional classifiers, MSAT had a clear performance margin, suggesting that casting CMM–ADR prediction as a heterogeneous graph problem can help leverage biomedical context.

### 4.2 Ablation study on MSAT components

Removal of any single component degraded performance relative to the full model (Table 3). Notably, removing HSP-Layer caused the largest drop, suggesting that hierarchical signal propagation is important for modeling multi-scale patterns from molecular binding to clinical phenotypes. Dropping ESA-Gate also led to a clear performance decline, supporting the importance of adaptive edge encoding for heterogeneous edge semantics. The model without HCI-Module saw a smaller yet consistent reduction (mostly in recall and MCC), reflecting HCI-Module’s role as a hub-calibrated inference mechanism.

The single-component variants further illustrate these roles. Variants with only ESA-Gate or only HSP-Layer performed better than only HCI-Module, but all three remained clearly inferior to the full MSAT model, and the differences in AUC relative to the full model were statistically significant (p<0.001) . Overall, the ablation results indicate that ESA-Gate, HSP-Layer and HCI-Module provide complementary benefits, and their combined use yields the strongest performance for CMM–ADR prediction in our evaluation.

### 4.3 Multi-dimensional robustness and generalization analysis

Beyond aggregate performance metrics, we carried out a multi-dimensional evaluation to assess MSAT’s external validity, robustness to degree-related bias and computational efficiency, which are all relevant for potential clinical deployment.

#### 4.3.1 External validity in cold-start scenarios

**FIGURE 5:**
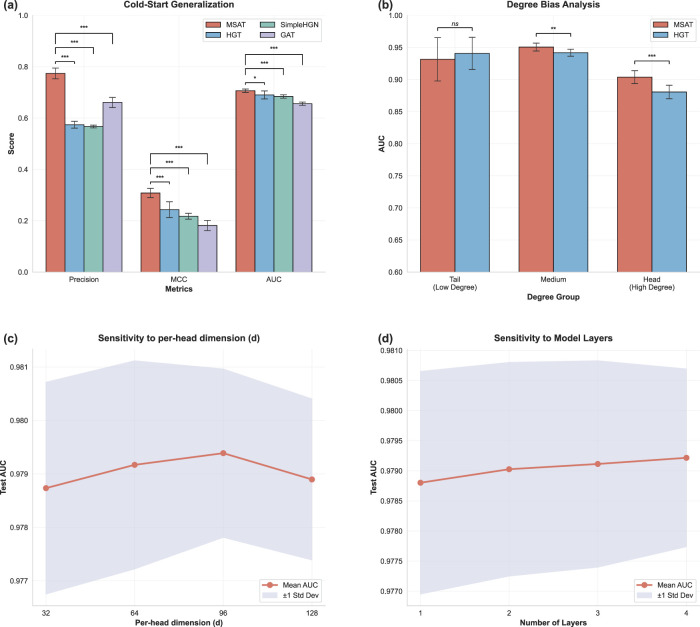
Multi-dimensional evaluation of MSAT. **(a)** Cold-start generalization on a literature-derived validation set in which 96.5% of CMM nodes are unseen during training. Bars show Precision, MCC and AUC for MSAT and benchmark GNNs, with significance annotations (*** p<0.001 , ** p<0.01 , * p<0.05 , ns not significant). **(b)** Degree-stratified AUC for Tail (low-degree), Medium and Head (high-degree) CMM nodes. **(c,d)** Sensitivity of test AUC to the per-head embedding dimension d (eight heads; total message size 8d ) and the number of layers L , respectively.

To test inductive generalization, we evaluated MSAT on an independent literature-derived dataset in which 96.5% of CMM nodes were unseen during training (Figure 5a). In this cold-start setting, baseline models showed marked drops in precision, whereas MSAT maintained substantially higher precision and MCC than all comparators, consistent with the use of heterogeneous pathways (e.g., compound–target–ADR) to support generalization beyond historical co-occurrence. On an additional 15% hold-out set from the FAERS-derived data (Supplementary Figure S1), the ROC curve (AUC = 0.936) further indicates stable out-of-sample discrimination within the same distribution.

#### 4.3.2 Robustness to degree bias

Biological and pharmacovigilance networks often follow heavy-tailed degree distributions, which can bias models towards high-degree entities. Degree-stratified analysis (Figure 5b) revealed a “head-hard” pattern in our CMM–ADR graph: high-degree CMMs (Head group) were more difficult to predict than low-degree ones (Tail group), likely due to noisy, poly-pharmacological interactions. MSAT alleviated this effect. Compared with HGT, MSAT achieved significantly higher AUC in the Head group (p<0.01) while maintaining comparable performance in the Tail group (ns), supporting the role of the Hub-Calibrated Inference (HCI) module in calibrating structural noise without degrading performance on long-tail medicines.

#### 4.3.3 Computational efficiency and stability

For practical use, predictive performance must be balanced against computational cost. The efficiency trade-off analysis (Supplementary Figure S2) shows test AUC as a function of training time per fold. Within the set of compared methods, MSAT provides a favorable accuracy–efficiency trade-off, achieving the highest AUC with moderate training time, whereas some lighter models are faster but less accurate and HGT is substantially slower. Finally, parameter sensitivity analyses (Figures 5c,d) indicate that MSAT is stable across a range of embedding sizes and layer depths: performance varies smoothly with narrow error bands, and the configuration used in the main experiments (Section 3.5.2) represents a strong accuracy–efficiency trade-off.

As an additional falsification test against shortcut learning via mechanistic Target–ADR links, we repeated training after removing all Target–ADR edges and after degree-preserving rewiring of Target–ADR edges. Performance remained within fold-to-fold variability (Supplementary Table S1), suggesting that the reported performance is not driven by a simple shortcut through Target–ADR relations.

### 4.4 Robustness to class imbalance

**FIGURE 6:**
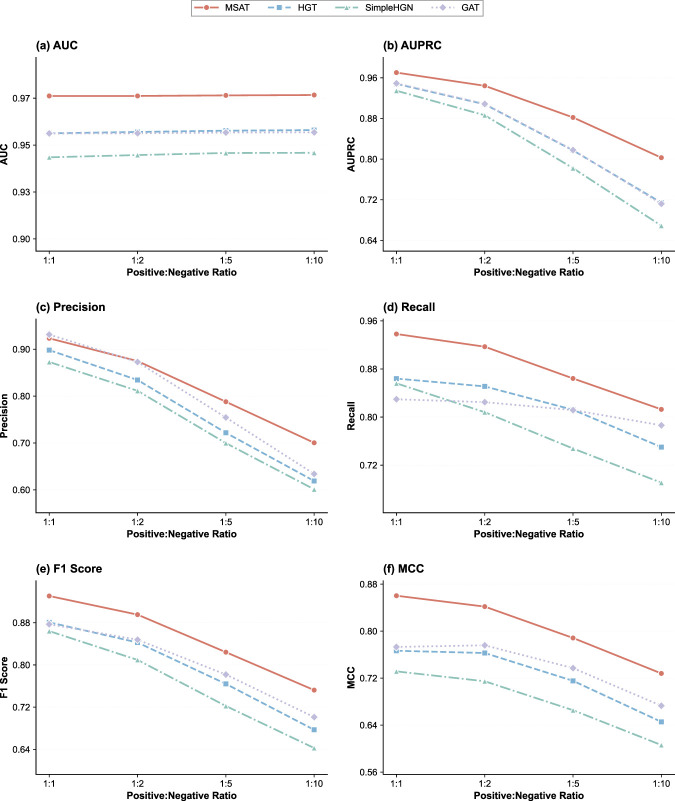
Effect of class imbalance on model performance. **(a–f)** AUC, AUPRC, precision, recall, F1-score and MCC for MSAT and three baseline models (HGT, Simple-HGN, GAT) under different positive:negative sampling ratios (1:1, 1:2, 1:5, 1:10) on the CMM–ADR prediction task.

Real-world pharmacovigilance data are highly imbalanced, with far fewer confirmed ADRs than non-events. To assess robustness under different imbalance levels, we varied the positive:negative sampling ratio from 1:1 to 1:10 and evaluated MSAT alongside three representative GNN baselines (HGT, Simple-HGN and GAT) on the CMM–ADR prediction task (Figure 6).

Across all ratios, MSAT maintained the highest AUC, AUPRC, F1-score and MCC among the compared models. As the proportion of negative samples increased, the performance of all methods declined, but MSAT showed a smaller loss than the baselines, particularly in AUPRC and MCC. This indicates that MSAT degrades more gracefully under increasing class imbalance and is better suited to the skewed CMM–ADR distributions encountered in practical pharmacovigilance settings.

While the above analysis evaluates robustness to test-set imbalance under balanced training, real-world pharmacovigilance must learn from inherently skewed data. We therefore performed an additional end-to-end imbalanced experiment where the training, validation, and test splits were all sampled at a 1:10 positive-to-negative ratio (Table 4). Under this setting, MSAT consistently ranked first and maintained a clear advantage over all baselines on AUC, AUPRC, and MCC (Table 4), indicating more reliable retrieval of rare adverse-reaction signals under end-to-end skew. Notably, R-GCN exhibited a high-precision but low-recall profile, suggesting an overly conservative strategy that would miss many true ADR signals in practice. In contrast, MSAT maintained a more balanced precision–recall trade-off, which is preferable in pharmacovigilance where both missed signals and false alarms carry tangible clinical costs. Traditional tabular classifiers degraded markedly under this skew, highlighting the advantage of graph-based models that can exploit relational structure when labels are sparse.

**TABLE 4:** End-to-end performance comparison of all models under 1:10 negative sampling.

Model	Precision	Recall	F1-score	AUC	AUPRC	MCC
Logistic Reg.	0.385 ± 0.022	0.488 ± 0.042	0.429 ± 0.006	0.819 ± 0.005	0.420 ± 0.007	0.368 ± 0.007
Random forest	0.496 ± 0.011	0.496 ± 0.009	0.496 ± 0.005	0.843 ± 0.005	0.483 ± 0.006	0.445 ± 0.006
XGBoost	0.567 ± 0.029	0.518 ± 0.020	0.540 ± 0.005	0.860 ± 0.004	0.555 ± 0.005	0.497 ± 0.008
GCN	0.569 ± 0.019	0.537 ± 0.024	0.552 ± 0.009	0.859 ± 0.006	0.582 ± 0.008	0.509 ± 0.009
GAT	0.578 ± 0.022	0.514 ± 0.019	0.543 ± 0.007	0.843 ± 0.006	0.554 ± 0.007	0.502 ± 0.008
R-GCN	**0.721** ± **0.016**	0.338 ± 0.016	0.460 ± 0.013	0.837 ± 0.005	0.521 ± 0.009	0.462 ± 0.010
HGT	0.547 ± 0.024	0.563 ± 0.020	0.554 ± 0.009	0.858 ± 0.005	0.567 ± 0.007	0.509 ± 0.010
HetGNN	0.296 ± 0.017	0.505 ± 0.041	0.372 ± 0.012	0.775 ± 0.007	0.283 ± 0.014	0.305 ± 0.015
Simple-HGN	0.622 ± 0.020	0.472 ± 0.009	0.537 ± 0.008	0.856 ± 0.005	0.576 ± 0.009	0.503 ± 0.010
MSAT	0.569 ± 0.028	**0.563** ± **0.028**	**0.565** ± **0.007**	**0.875** ± **0.005**	**0.603** ± **0.008**	**0.523** ± **0.008**

Values are reported as mean ± standard deviation over stratified 10-fold cross-validation.

Bold values indicate the best performance for each evaluation metric.

### 4.5 Validation of predicted results

To assess the plausibility of MSAT’s ranked predicted results, we first reviewed the Top 15 highest-confidence predictions using the protocol in Section 3.5.6 (Table 5). Overall, 13 of 15 (86.7%) had support from either database verification or literature/mechanistic evidence. Because this assessment is defined relative to an incomplete labeled edge list (rather than a claim of real-world novelty), some clinically recognized effects can appear in the prediction list if they are unlabeled under FAERS-driven extraction and conservative term normalization/filtering. For example, Changshan (*Dichroa febrifuga* Lour.)–vomiting is supported by the literature (Sun et al., 2022), yet it was not captured as a labeled positive under our curated supervision; we therefore interpret this instance as evidence of label incompleteness rather than as a “new” finding. To mitigate the selection bias inherent to a Top- K -only analysis, we further evaluated two *uniformly randomly sampled* strata of additional predictions. In the mid-confidence stratum (ranks 21–100), 8 of 15 predictions (53.3%) were supported by at least one validation channel (Supplementary Table S5). In the lower-confidence stratum (ranks 101–200), 3 of 15 predictions (20.0%) were supported (Supplementary Tables S7). This decline across strata is consistent with score-based prioritization: higher-ranked predictions are enriched for externally supported associations, whereas lower-ranked predictions increasingly reflect uncertain hypotheses. Mechanistic context can further aid interpretation when direct CMM-level evidence is sparse. For example, prior evidence suggests that salicylate constituents related to Ciwujia may contribute to tinnitus (Sheppard et al., 2014), offering a plausible rationale for the predicted phenotype. Together, these analyses suggest that MSAT can help prioritize candidate CMM–ADR signals for downstream review, including associations not captured as labeled positives in our curated supervision.

**TABLE 5:** Predicted CMM–ADR results: external evidence check for the Top 15 highest-confidence predictions.

CMM Pinyin	Scientific name	Predicted ADR (MedDRA PT)	MSAT score (%)	Database verified	Mechanistic support
Huzhang	*Polygonum cuspidatum* Sieb. et Zucc.	Vomiting	99.943	Yes	${\text{Yes}}^{a}$
Changshan	*Dichroa febrifuga* Lour.	Vomiting	99.941	No	${\text{Yes}}^{b}$
Hongjingtian	*Rhodiola crenulata* (Hook.f. et Thomson) H.Ohba	Palpitations	99.893	No	No mechanistic evidence
Muzei	*Equisetum hyemale* L.	Vomiting	99.890	Yes	${\text{Yes}}^{c}$
Luole	*Ocimum basilicum* L.	Acute pulmonary oedema	99.868	Yes	No mechanistic evidence
Aiye	*Artemisia argyi* Levl. et Vaniot	Drug-induced liver injury	99.710	Yes	${\text{Yes}}^{e,f}$
Ezhu	*Curcuma phaeocaulis* Valeton	Dermatitis	99.593	Yes	${\text{Yes}}^{g}$
Niubanggen	*Arctium lappa* L.	Acute pulmonary oedema	99.473	Yes	${\text{Yes}}^{h}$
Shanglu	*Phytolacca acinosa* Roxb.	Gastric haemorrhage	99.393	Yes	${\text{Yes}}^{i,j,k}$
Bajiao Huixiang	*Illicium verum* Hook.f.	Pulmonary embolism	99.153	Yes	No mechanistic evidence
Yuanhua	*Daphne genkwa* Sieb. et Zucc.	Small intestinal haemorrhage	98.843	Yes	${\text{Yes}}^{l}$
Ciwujia	*Eleutherococcus senticosus* (Rupr. et Maxim.) Maxim.	Tinnitus	98.742	Yes	${\text{Yes}}^{m}$
Hei Shengma	*Cimicifuga racemosa* (L.) Nutt.	Dizziness	98.291	No	${\text{Yes}}^{n}$
Liangmianzhen	*Zanthoxylum nitidum* (Roxb.) DC.	Hepatitis	98.198	No	No mechanistic evidence
Xishu	*Camptotheca acuminata* Decne.	Tremor	97.923	Yes	${\text{Yes}}^{o}$

Sorted by MSAT, score in descending order. Mechanistic literature support: ^a^(Liu et al., 2018); ^b^(Sun et al., 2022); ^c^(Mishra et al., 2015); ^e^(Liu et al., 2017); ^f^(Gong et al., 2011); ^g^(Kong et al., 2023); ^h^(Solomons and Cochrane, 1984); ^i^(Hung and Hsu, 1998); ^j^(Ogawa et al., 1981); ^k^(Hung, 2005); ^l^(Chen et al., 2016); ^m^(Sheppard et al., 2014); ^n^(Oleszczuk et al., 2008); ^o^(Woods, 1879).

#### 4.5.1 Interpretability case study (path tracing)

To provide a deeper, clinician-facing explanation of a representative prediction, we traced an explicit multi-hop path in the curated heterogeneous graph for the CMM–ADR pair Zhǐ Shí (*Citrus aurantium* L.) → diarrhoea. This path is presented as an illustrative, *post hoc* mechanistic rationale available in the curated graph (rather than as a uniquely identified explanation from attention weights), and is intended to support hypothesis generation. A salient path highlights a plausible mechanism-oriented chain via nobiletin (PubChem CID 72344) and the transporter ABCG2 (BCRP), linking a CMM constituent to a target with GI-relevant adverse-event mechanisms discussed in prior literature (Pick et al., 2011; Tao and Chityala, 2021). This case study is intended for interpretability and hypothesis generation rather than causal attribution (Supplementary Figure S5).

#### 4.5.2 TCM functional system mapping validation

A primary objective of this study was to ensure computational predictions are interpretable within TCM clinical practice. We therefore applied the mapping framework established in Section 3.4.2 to translate the 15 high-confidence predicted MedDRA-coded ADRs (listed in Table 5) into the 16 TCM functional system categories. The results are presented in Table 6.

**TABLE 6:** TCM functional system mapping validation results.

CMM Pinyin	Scientific name	Predicted ADR (MedDRA PT)	TCM system mapping
Huzhang	*Polygonum cuspidatum* Sieb. et Zucc.	Vomiting	Stomach
Changshan	*Dichroa febrifuga* Lour.	Vomiting	Stomach
Hongjingtian	*Rhodiola crenulata* (Hook.f. et Thomson) H.Ohba	Palpitations	Heart
Muzei	*Equisetum hyemale* L.	Vomiting	Stomach
Luole	*Ocimum basilicum* L.	Acute pulmonary oedema	Lung+Qi-Blood-Fluid
Aiye	*Artemisia argyi* Levl. et Vaniot	Drug-induced liver injury	Liver+Qi-Blood-Fluid
Ezhu	*Curcuma phaeocaulis* Valeton	Dermatitis	Body Surface
Niubanggen	*Arctium lappa* L.	Acute pulmonary oedema	Lung+Qi-Blood-Fluid
Shanglu	*Phytolacca acinosa* Roxb.	Gastric haemorrhage	Stomach+Qi-Blood-Fluid
Bajiao Huixiang	*Illicium verum* Hook.f.	Pulmonary embolism	Lung+Qi-Blood-Fluid
Yuanhua	*Daphne genkwa* Sieb. et Zucc.	Small intestinal haemorrhage	Small Intestine+Qi-Blood-Fluid
Ciwujia	*Eleutherococcus senticosus* (Rupr. et Maxim.) Maxim.	Tinnitus	Kidney
Hei Shengma	*Cimicifuga racemosa* (L.) Nutt.	Dizziness	Liver
Liangmianzhen	*Zanthoxylum nitidum* (Roxb.) DC.	Hepatitis	Liver+Qi-Blood-Fluid
Xishu	*Camptotheca acuminata* Decne.	Tremor	Body Surface

Sorted by MSAT, score in descending order.

Overall, the mapped functional systems were consistent with common Zang–Fu interpretations for the predicted phenotypes. ADRs with clear organ-system manifestations (e.g., vomiting) were mapped to the expected systems (e.g., Stomach).

Several mappings also reflected widely taught associations in traditional diagnostics: tinnitus was mapped to the Kidney system, consistent with Zang–Fu doctrine describing the Kidney’s relationship with the ears (Unschuld and Tessenow, 2011); dizziness was mapped to the Liver system, in line with commonly referenced interpretations such as liver-yang rising (Unschuld and Tessenow, 2011).

These results indicate that MSAT outputs can be rendered in a clinician-facing functional vocabulary, improving interpretability for TCM-oriented pharmacovigilance.

## 5 Discussion

In this study, we propose MSAT, a clinically aligned heterogeneous graph learning framework for predicting adverse reactions associated with Chinese Materia Medica (CMM). MSAT integrates multi-source pharmacological and safety data into a heterogeneous CMM–Compound–Target–ADR graph and combines three tailored components—ESA-Gate, HSP-Layer, and HCI-Module—designed for evidence-rich pharmacovigilance graphs. Across stratified 10-fold cross-validation, MSAT consistently outperformed a range of baseline models, including traditional machine-learning methods, homogeneous GNNs, and representative heterogeneous GNN baselines. Beyond aggregate performance, MSAT showed robust cold-start generalization, resilience to degree-related bias and class imbalance, and outputs that can be expressed in TCM functional system terms. Importantly, MSAT is intended for risk prioritization and hypothesis generation rather than causal attribution; its outputs should be interpreted in conjunction with clinical assessment and established pharmacovigilance workflows. We next discuss the implications of these findings, the contribution of the proposed architecture, and limitations that suggest directions for future work. Additional robustness and sensitivity analyses are reported in Supplementary Tables S1–S3 and Supplementary Figures S1, S3–S4.

### 5.1 Contribution of the MSAT architecture

A central motivation of this work was that accurate CMM–ADR prediction requires models that reflect both the semantic heterogeneity of biomedical associations and the multi-scale structure of pharmacological mechanisms. The ablation study supports this view. In particular, removing HSP-Layer led to the largest performance drop, consistent with the need to model hierarchical and non-linear transduction from molecular binding to clinical phenotypes. As a hierarchical signal propagation mechanism, HSP-Layer appears important for capturing the long-range dependencies linking molecular components to clinical outcomes.

ESA-Gate’s contribution suggests that evidence-bearing CMM–ADR edges should be treated differently from binary biological links. By injecting edge evidence as an attention bias, ESA-Gate helps preserve quantitative FAERS signals that might otherwise be diluted by relation-level projections. Removing ESA-Gate reduced discrimination and recall, indicating that uniform transformations across edge types can underutilize the rich edge attributes. In our graph, CMM–ADR edges carry multi-dimensional attributes derived from FAERS statistics, literature evidence, and structural context, which are fundamentally different from binary biological edges. ESA-Gate allows the model to adaptively balance non-linear transformation and direct information preservation, supporting detection of subtle but clinically meaningful risk signals.

HCI-Module provided a complementary benefit. Although removing HCI-Module had a smaller effect than removing HSP-Layer or ESA-Gate, the model’s performance still declined (particularly in recall and MCC). This outcome aligns with HCI-Module’s role as a hub-calibrated inference mechanism combining multiple scoring functions, capturing both symmetric similarity and directional (asymmetric) association patterns. The degree-aware weighting in HCI-Module further calibrates predictions for both high- and low-degree nodes. Overall, the ablation results suggest that MSAT’s performance advantage arises from the combined action of these three components rather than from any single module in isolation.

### 5.2 Clinical and pharmacological implications

From a clinical perspective, an important contribution of this work is the mapping of predicted MedDRA-coded ADRs into sixteen TCM functional systems. Conventional pharmacovigilance models output signals in regulatory terminology that may not align with how TCM clinicians conceptualize organ systems and syndromes. By translating ADR predictions into categories based on Zang–Fu organs and key functional systems (such as Qi-Blood-Fluid and meridian systems), MSAT produces outputs that can be more directly integrated into TCM decision-making.

The case analyses illustrate this potential. For example, predicted vomiting events for specific CMMs were correctly assigned to the Stomach system, and tinnitus was associated with the Kidney system, consistent with the classical principle that the Kidney “opens into the ears.” Similarly, dizziness mapped to the Liver system, aligning with patterns such as liver-yang rising in TCM theory. These examples suggest that, when MSAT identifies a potential ADR, the corresponding TCM system mapping can help clinicians interpret the signal within their diagnostic framework, guiding syndrome differentiation, formula adjustments, and targeted monitoring of high-risk organ systems.

Pharmacologically, MSAT recovered both well-documented and literature-reported CMM–ADR associations and also produced high-confidence predicted signals for downstream review. This pattern suggests that heterogeneous pathways (e.g., compound–target–ADR chains) carry meaningful predictive information. The cold-start evaluation, in which most CMMs were unseen during training, is particularly relevant. In this setting, MSAT maintained high precision and MCC, suggesting that it can extrapolate risk patterns based on chemical and network context rather than relying solely on historical co-occurrence. This capability may be important for proactive safety assessment of emerging CMMs and formula components.

From a pharmacovigilance operations standpoint, MSAT is best positioned as a signal-triage and prioritization layer, rather than a replacement for standard disproportionality screening. In a periodic monitoring setting, MSAT can produce a ranked list of candidate CMM–ADR signals that are supported by spontaneous reporting evidence and coherent with mechanistic paths in the heterogeneous graph (e.g., compound–target–ADR). Such rankings can help safety teams prioritize manual case review, targeted literature assessment, and downstream pharmacoepidemiological or experimental follow-up, particularly for long-tail CMMs where historical evidence is sparse and rule-based screening is less informative. Compared with marginal-count disproportionality analyses, MSAT incorporates FAERS-derived reporting evidence (log-transformed case report counts and provenance) as edge features while jointly modeling mechanistic connectivity, enabling context-aware prioritization under confounding and sparsity.

### 5.3 Methodological implications for graph-based pharmacovigilance

Methodologically, this work contributes to a growing line of research applying graph representation learning to pharmacovigilance. Existing heterogeneous GNNs such as R-GCN (Schlichtkrull et al., 2018), HGT (Hu et al., 2020), and Simple-HGN (Lv et al., 2021) provide strong baselines, but they are primarily designed around relation-type–specific message passing on graph topology, with limited emphasis on high-dimensional edge attributes and degree-aware calibration. These results suggest that, in settings where edges carry semantically rich features and where degree distributions are highly skewed, explicit edge encoders and late fusion mechanisms can yield measurable gains.

Our multi-dimensional evaluation further highlights the importance of going beyond average test metrics. Degree-stratified analysis revealed a “head-hard” phenomenon in the CMM–ADR graph: high-degree CMMs were more difficult to predict, likely due to noisy and heterogeneous interaction patterns. MSAT improved performance in this head group without sacrificing accuracy on long-tail CMMs, suggesting a pathway to fairer and more robust graph models in other biomedical domains with similar degree distributions. Likewise, robustness across class-imbalance ratios suggests that MSAT is relatively stable under the skewed positive–negative ratios that characterize many real-world safety datasets.

Although this study focuses on CMM–ADR prediction, the underlying ideas of ESA-Gate, HSP-Layer and HCI-Module are more general. Similar architectures could be explored for tasks such as drug–drug interaction (DDI) prediction, target deconvolution, or adverse event forecasting in other complementary and integrative medicine systems, where heterogeneous evidence and theory-specific clinical frameworks coexist.

### 5.4 Limitations and future work

Several limitations should be acknowledged. First, the ground-truth CMM–ADR associations are derived primarily from FAERS and other spontaneous reporting sources, supplemented by expert-curated literature. Such data are prone to under-reporting, stimulated reporting, indication and channeling bias, co-medication confounding, and duplicate or incomplete reports, and they do not provide causal confirmation (Harpaz et al., 2012). Moreover, FAERS lacks exposure denominators, so the predicted scores should be interpreted as relative signal strength for prioritization rather than incidence or absolute risk. Co-medication and indication-related confounding are particularly salient in FAERS. We do not perform causal adjustment in this study. As a report-level sensitivity analysis, we restricted to monotherapy-dominant TCM-containing reports (defined as reports with exactly one unique drug in the exported table). Under this proxy definition, the strongest CMM–ADR report-count edges remained highly stable relative to the full set (Supplementary Figure S4), suggesting that the most prominent signals are not solely driven by polypharmacy. In addition, a single standardized CMM name can correspond to heterogeneous commercial preparations and formulations with variable constituent profiles in FAERS, which can dilute attribution and introduce label noise. Finally, curated resources used to instantiate mechanistic edges (e.g., Target–ADR) may be semantically correlated with FAERS-derived signals. We interpret this as real-world knowledge overlap and residual confounding rather than cross-validation leakage under our fold-wise, bidirectional test-edge removal protocol. Second, our supervised evaluation relies on type-constrained negative sampling, where negatives represent unobserved CMM–ADR pairs rather than confirmed non-associations; while standard in link prediction, this setting may not fully reflect real-world decision thresholds. For comparability, we retain uniform type-constrained negatives in the main protocol and additionally report a mechanism-aware hard-negative sensitivity analysis (Supplementary Table S3). More principled hard-negative construction that incorporates richer exposure or covariate information remains an important direction for future work. Third, because FAERS often lacks reliable dosage and formulation details for CMM products, MSAT currently focuses on hazard identification (signal detection) rather than dose-response or risk characterization. We encoded FAERS evidence primarily via report-count–based features; incorporating classical disproportionality metrics (e.g., ROR/PRR) will be explored in future work to further characterize signal strength under confounding. Future work should incorporate complementary data sources (e.g., electronic health records, prospective registries, or active surveillance systems) and temporal analyses to strengthen validation and enable more clinically actionable risk stratification.

The TCM functional system mapping is based on expert-defined rules and authoritative references, and was evaluated using a limited number of case studies. The current mapping operates at the level of organ and functional systems rather than full syndrome patterns. Constrained by the granularity of the source data, it does not account for inter-individual variability, formula composition, or dosing regimens. Extending the mapping to finer-grained TCM entities (e.g., syndrome patterns and meridians) and validating it in clinical cohorts will be important steps toward routine use in practice.

Regarding interpretability, although MSAT improves clinical alignment at the level of outputs, the internal reasoning process of the model remains only partially transparent. This work did not systematically apply *post hoc* explanation techniques for GNNs, and therefore cannot fully characterize which specific pathways (e.g., particular compounds, targets, or subgraphs) drive individual predictions. Future research could integrate pathway-level explanation methods or attention-based path-tracing techniques to highlight the most influential components in each CMM–ADR association and to generate mechanistic hypotheses.

Finally, the present study focuses on single CMMs. In clinical practice, CMMs are most often used as part of multi-CMM formulas following principles such as Jun–Chen–Zuo–Shi (sovereign, minister, assistant, and courier), and interactions among ingredients can alter both efficacy and safety. Extending the heterogeneous graph to include formula nodes and explicit formula–CMM relationships would enable MSAT-like architectures to predict ADRs at the formula level and to study how different combinations modify risk. Incorporating patient-level covariates and temporal information would further support personalized and dynamic safety assessment.

Overall, this work illustrates how clinically aligned machine learning can connect computational pharmacovigilance with traditional medicine frameworks. While further validation and refinement are needed, especially in prospective and formula-level settings, our results suggest a practical template: combining semantic edge encoding, multi-scale representation learning, and theory-grounded clinical mapping can move models beyond black-box prediction toward outputs that are more interpretable in clinical context. Such alignment between data-driven modeling and domain knowledge may help support translation of safety informatics into integrative medicine practice.

## 6 Conclusion

We have presented MSAT, a heterogeneous graph neural network framework explicitly designed for the safety assessment of Chinese Materia Medica. MSAT integrates multi-scale biomedical data with tailored architectural components to model the pathways from medicinal materials to clinical adverse reactions. In stratified 10-fold cross-validation, the framework demonstrated superior discriminative performance compared to both shallow baselines and state-of-the-art heterogeneous graph models.

Beyond overall accuracy, MSAT recovered a high proportion of literature-supported CMM–ADR associations and proposed additional pharmacologically plausible risks. The framework also links predicted adverse reactions to sixteen TCM functional systems, which allows the outputs to be understood within familiar Zang–Fu concepts and related functional categories.

Taken together, these results show that MSAT provides a practical tool for modern TCM pharmacovigilance. It offers a foundation grounded in data for prioritizing safety signals, supports hypothesis generation about potential mechanisms and organ systems, and facilitates the integration of computational drug-safety research with traditional clinical decision-making.

## Data Availability

Publicly available datasets were analyzed in this study. This data can be found here: Zenodo, identification number 10.5281/zenodo.17933842 (https://zenodo.org/records/17933842); GitHub, direct URL https://github.com/BowenShiGDPU/MSAT; FDA Adverse Event Reporting System (FAERS), direct URL: https://www.fda.gov/drugs/surveillance/fdas-adverse-event-reporting-system-faers.
