# Supplementary material for: MSAT: a FAERS-informed heterogeneous graph neural network for pharmacovigilance prediction of Chinese materia medica–associated adverse drug reactions
Source: Front Pharmacol. 2026 Feb 26;17:1774128. doi: 10.3389/fphar.2026.1774128 (PMC12979427; doi:10.3389/fphar.2026.1774128)
Supplement: Supplementary file 1 [file DataSheet1.pdf]

Additional File 1

1 FULL HYPERPARAMETER CONFIGURATIONS FOR ALL BASELINE MODELS

This document provides detailed hyperparameter configurations for all baseline models used in the comparative evaluation. All models were trained with consistent data splits and negative sampling strategies to ensure fair comparison.

1.1 Graph Neural Network Baselines

1.1.1 GCN (Graph Convolutional Network)

- Hidden dimension: 576
- Number of layers: 3
- Activation: ReLU
- Dropout rate: 0.18
- Learning rate: 4×10^{-4}
- Weight decay: 1×10^{-5}
- Batch size: 512
- Optimizer: AdamW
- Maximum epochs: 1000
- Early stopping patience: 100 epochs
- Gradient clipping: max norm 1.0
- Learning rate scheduler: ReduceLROnPlateau (factor 0.6, patience 15)

1.1.2 GAT (Graph Attention Network)

- Hidden dimension: 576
- Number of layers: 3
- Number of attention heads: 8
- Activation: ReLU
- Dropout rate: 0.18
- Learning rate: 4×10^{-4}
- Weight decay: 1×10^{-5}
- Batch size: 512
- Optimizer: AdamW
- Maximum epochs: 1000
- Early stopping patience: 100 epochs
- Gradient clipping: max norm 1.0
- Learning rate scheduler: ReduceLROnPlateau (factor 0.6, patience 15)

1.1.3 R-GCN (Relational Graph Convolutional Network)

- Hidden dimension: 576
- Number of layers: 3
- Activation: ReLU
- Dropout rate: 0.18
- Learning rate: 4×10^{-4}
- Weight decay: 1×10^{-5}
- Batch size: 512
- Optimizer: AdamW
- Maximum epochs: 1000
- Early stopping patience: 100 epochs
- Gradient clipping: max norm 1.0
- Learning rate scheduler: ReduceLROnPlateau (factor 0.6, patience 15)

1.1.4 HGT (Heterogeneous Graph Transformer)

- Hidden dimension: 576
- Number of layers: 3
- Number of attention heads: 8
- Activation: ReLU
- Dropout rate: 0.18
- Learning rate: 4×10^{-4}
- Weight decay: 1×10^{-5}
- Batch size: 512
- Optimizer: AdamW
- Maximum epochs: 1000
- Early stopping patience: 100 epochs
- Gradient clipping: max norm 1.0
- Learning rate scheduler: ReduceLROnPlateau (factor 0.6, patience 15)

1.1.5 Simple-HGN (Simple Heterogeneous Graph Network)

- Hidden dimension: 576
- Number of layers: 3
- Number of attention heads: 8
- Activation: ReLU
- Dropout rate: 0.18
- Learning rate: 4×10^{-4}
- Weight decay: 1×10^{-5}
- Batch size: 512
- Optimizer: AdamW

- Maximum epochs: 1000
- Early stopping patience: 100 epochs
- Gradient clipping: max norm 1.0
- Learning rate scheduler: ReduceLROnPlateau (factor 0.6, patience 15)

1.1.6 HetGNN (Heterogeneous Graph Neural Network)

- Hidden dimension: 576
- Number of layers: 3
- Activation: ReLU
- Dropout rate: 0.18
- Learning rate: 4×10^{-4}
- Weight decay: 1×10^{-5}
- Batch size: 512
- Optimizer: AdamW
- Maximum epochs: 1000
- Early stopping patience: 100 epochs
- Gradient clipping: max norm 1.0
- Learning rate scheduler: ReduceLROnPlateau (factor 0.6, patience 15)

1.2 Traditional Machine Learning Baselines

1.2.1 Logistic Regression

- Regularization: L2
- Regularization strength (C): 1.0
- Solver: liblinear
- Maximum iterations: 1000
- Tolerance: 1×10^{-4}

1.2.2 Random Forest

- Number of trees: 100
- Maximum depth: None
- Minimum samples split: 2
- Minimum samples leaf: 1
- Maximum features: sqrt
- Random state: 42

1.2.3 XGBoost

- Number of estimators: 100
- Maximum depth: 6
- Learning rate: 0.1
- Subsample: 0.8

- Column sample by tree: 0.8
- Gamma: 0
- Minimum child weight: 1
- Random state: 42

2 TRAINING PROTOCOL

All models were trained using the following common protocol:

- Data splitting: Stratified 10-fold cross-validation
- Train/validation/test split: 81:9:10 (within each fold)
- Negative sampling: Type-constrained (1:1 for main experiments, 1:10 for end-to-end imbalanced setting)
- Evaluation metric for early stopping: Validation AUC
- Model selection: Best checkpoint based on validation AUC

3 COMPUTATIONAL ENVIRONMENT

All experiments were conducted using:

- Python version: 3.8+
- PyTorch version: 1.12+
- CUDA version: 11.6+ (for GPU acceleration)
- Hardware: NVIDIA GPUs with at least 8GB memory

4 SUPPLEMENTARY TABLES

4.0.0.1 Interpretation.

If MSAT were primarily exploiting Target–ADR edges as a shortcut, removing (A1) or rewiring those edges while preserving degree statistics (A2) would be expected to substantially degrade performance. Instead, performance remained within fold-to-fold variability across A0/A1/A2, suggesting that the reported performance is not driven by a simple shortcut through Target–ADR mechanistic edges.

4.0.0.2 Experimental controls.

All settings used the same 10-fold splits, identical training hyperparameters and random seeds, and differed only in the Target–ADR edge structure (A0/A1/A2).

Table S1. Target–ADR mechanistic-edge falsification tests (10-fold CV). We compare MSAT performance under (A0) the original graph, (A1) removing all Target–ADR edges, and (A2) degree-preserving rewiring of Target–ADR edges (shuffling endpoints while preserving per-node degree distributions). In all settings, held-out test positive CMM–ADR edges are removed from the graph before training/inference (inductive evaluation), in both directions, with edge attributes deleted synchronously. Values are mean ± std over 10 folds.

Setting	AUC	AUPRC	Precision	Recall	F1	MCC
A0 Baseline	0.9791±0.0017	0.9765±0.0025	0.9271±0.0042	0.9336±0.0058	0.9303±0.0037	0.8601±0.0068
A1 Remove Target–ADR	0.9787±0.0019	0.9761±0.0027	0.9266±0.0064	0.9308±0.0064	0.9287±0.0041	0.8571±0.0075
A2 Rewire Target–ADR	0.9791±0.0018	0.9764±0.0029	0.9271±0.0055	0.9355±0.0044	0.9313±0.0034	0.8620±0.0062

4.0.0.3 Interpretation (FAERS label noise sensitivity).

FAERS-derived associations are pharmacovigilance *signals* rather than causal confirmations, and low-count pairs are often noisier. When restricting positives to higher-count FAERS associations (larger t), MSAT performance is stable and tends to improve across AUC/AUPRC/F1, consistent with increased signal-to-noise at higher report-count thresholds. Because this analysis uses FAERS-only positives and rebuilds the sampled evaluation set for each threshold, absolute metrics should be interpreted within this sensitivity setting. The primary conclusion is the monotonic robustness trend as the minimum count threshold increases.

4.0.0.4 Experimental controls.

For each threshold, we used a fixed random seed and the same model architecture and training hyperparameters. The only change is the FAERS-only positive definition (and the corresponding CMM–ADR edges and their reverse edges in the graph) induced by the report-count threshold.

Table S2. Sensitivity analysis: restricting FAERS-derived positives by minimum report count. We restrict positive CMM–ADR edges to FAERS-only associations (`has_faers`=1) with report count $\geq t$ for $t \in \{1, 3, 5, 10\}$. For each threshold, we rebuild the CMM–ADR edge set in the graph (including its reverse edges) and re-run 10-fold cross-validation under the main (balanced 1:1) training protocol with inductive test-edge removal. Values are mean $\pm$ std over 10 folds. This analysis evaluates robustness under stricter FAERS-only supervision and is not intended for direct numerical comparison with the mixed-source (FAERS+literature) setting.

FAERS min count	AUC	AUPRC	Precision	Recall	F1	MCC
$t \geq 1$	0.8700±0.0038	0.8833±0.0054	0.8066±0.0094	0.7796±0.0076	0.7928±0.0050	0.5930±0.0105
$t \geq 3$	0.8779±0.0054	0.8924±0.0059	0.8157±0.0151	0.7962±0.0116	0.8057±0.0073	0.6163±0.0160
$t \geq 5$	0.8821±0.0071	0.8964±0.0049	0.8152±0.0132	0.8014±0.0137	0.8081±0.0072	0.6198±0.0150
$t \geq 10$	0.8900±0.0044	0.9047±0.0061	0.8280±0.0133	0.8062±0.0108	0.8169±0.0086	0.6389±0.0171

4.0.0.5 Interpretation (negative-sampling robustness).

Sampled negatives in pharmacovigilance link prediction represent unobserved pairs rather than confirmed non-associations. To stress-test robustness beyond uniform negatives, we construct mechanism-aware hard negatives by sampling ADR candidates from the CMM's mechanistic neighborhood, while filtering out all known positives to mitigate false negatives. MSAT maintained strong discrimination under this harder negative set, suggesting that the reported conclusions are robust to a more challenging negative-sampling strategy.

4.0.0.6 Experimental controls.

All settings used the same FAERS-only positive definition and the same training protocol. Negatives were replaced by mechanism-aware candidates when available (with uniform fallback otherwise).

Table S3. Sensitivity analysis: mechanism-aware hard negatives (FAERS-only, 1:1). Positives are restricted to FAERS-only CMM–ADR edges (`has_faers`=1). Negatives are generated by fixing the CMM and sampling ADRs from a mechanism-aware candidate pool constructed from the CMM's target neighborhood, while excluding all known positives for that CMM (FAERS+literature) to mitigate false negatives. If the candidate pool is empty, we fall back to uniform type-constrained sampling. Values are mean $\pm$ std over 10 folds under the main (balanced 1:1) training protocol with inductive test-edge removal.

Setting	AUC	AUPRC	Precision	Recall	F1	MCC
FAERS-only + hard negatives	0.8894±0.0045	0.9020±0.0053	0.8212±0.0087	0.8056±0.0111	0.8133±0.0066	0.6304±0.0107

4.0.0.7 Implementation note (avoiding ID leakage).

Feature ablation is implemented using *zero vectors* rather than random vectors. Random vectors can inadvertently act as node identifiers and inflate performance under pair-random CV. Zeroing removes semantic content while preserving graph topology and message passing.

Table S4. Data-source-level ablation study. We quantify the contribution of major heterogeneous information sources by selectively ablating either (i) *semantic node features* (by setting the corresponding node embeddings to zero vectors), or (ii) *structural mechanistic relations* (by removing a specific edge type) in the heterogeneous graph. All values are mean ± std over 10-fold cross-validation under the main (balanced 1:1) protocol.

Variant	AUC	AUPRC	Precision	Recall	F1	MCC
baseline	0.9790±0.0015	0.9770±0.0018	0.9270±0.0045	0.9330±0.0040	0.9300±0.0030	0.8600±0.0055
no_compound_feat	0.9680±0.0020	0.9650±0.0025	0.9120±0.0055	0.9220±0.0050	0.9170±0.0042	0.8350±0.0080
no_target_feat	0.9550±0.0028	0.9510±0.0032	0.8950±0.0070	0.9070±0.0062	0.9010±0.0055	0.8030±0.0105
no_ppi_edges	0.9700±0.0018	0.9670±0.0022	0.9150±0.0052	0.9240±0.0048	0.9195±0.0038	0.8400±0.0072
no_adr_feat	0.9480±0.0032	0.9430±0.0038	0.8850±0.0078	0.9000±0.0068	0.8925±0.0060	0.7860±0.0115
no_target_adr_edges	0.9710±0.0018	0.9680±0.0022	0.9180±0.0052	0.9260±0.0048	0.9220±0.0038	0.8450±0.0075
no_herb_feat	0.9520±0.0030	0.9480±0.0035	0.8900±0.0075	0.9050±0.0065	0.8975±0.0058	0.7960±0.0110
no_compound_full	0.9450±0.0035	0.9400±0.0042	0.8800±0.0085	0.8950±0.0072	0.8875±0.0065	0.7760±0.0125
no_adr_full	0.9350±0.0040	0.9280±0.0048	0.8650±0.0095	0.8850±0.0080	0.8750±0.0072	0.7510±0.0140

Table S5. Validation of randomly sampled predictions from rank 21–100. We uniformly randomly sampled 15 predicted CMM–ADR pairs from the model's rank 21–100 list (mid-confidence stratum), after excluding labeled positive edges used for supervised evaluation. Each pair was evaluated using the same two-channel protocol as the Top-15 analysis: **Database verification** (TCMTOX concordance) and **Literature/mechanistic support**. A prediction is counted as *validated* if *either* channel provides support (logical OR), consistent with pharmacovigilance signal triage.

CMM Pinyin	Scientific Name	Predicted ADR (MedDRA PT)	MSAT Score (%)	Database Verified	Mechanistic Support
HeShouWu	*Polygonum multiflorum* Thunb.	Hepatic function abnormal	97.654	Yes	Yes[a]
Hongchezhou-cao	*Trifolium pratense* L.	Drug-induced liver injury	97.312	Yes	Yes[b]
Hehuanpi	*Albizia julibrissin* Durazz.	Delirium	96.985	No	No
Yanhusuo	*Corydalis yanhusuo* W.T.Wang	Somnolence	96.540	Yes	Yes[c]
Hongjingtian	*Rhodiola crenulata* (Hook.f. et Thomson) H.Ohba	Dizziness	96.220	No	Yes[d]
Jixingzi	*Impatiens balsamina* L.	Dizziness	95.890	No	No
Daji	*Euphorbia pekinensis* Rupr.	Vomiting	95.550	No	No
Longkui	*Solanum nigrum* L.	Nausea	95.125	No	Yes[e]
Suoyang	*Cynomorium songaricum* Rupr.	Vomiting	94.880	No	Yes[f]
Wuzhuyu	*Euodia rutaecarpa* (Juss.) Benth.	Hepatic function abnormal	94.560	Yes	Yes[g]
Tianhuafen	*Trichosanthes kirilowii* Maxim.	Vomiting	94.210	Yes	No
Pipaye	*Eriobotrya japonica* (Thunb.) Lindl.	Urethral pain	93.950	No	No
Danshen	*Salvia miltiorrhiza* Bunge	Abdominal pain	93.660	No	No
Baiguo	*Ginkgo biloba* L.	Ear discomfort	93.320	No	No
Zhugenqi	*Disporopsis fuscopicta* Hance	Vomiting	93.150	No	No

Mechanistic literature support: [a](Li et al., 2019), [b](Engelhardt and Riedl, 2008), [c](Wang and Mantsch, 2012), [d](Mao et al., 2015), [e](Roelen et al., 2024), [f](Zhang et al., 2024), and [g](Cai et al., 2014).

Table S6. TCM functional system mapping for the randomly sampled rank 21–100 predictions. We report the corresponding clinician-facing functional system mapping (same rules as in the main text) for the 15 randomly sampled predictions in Table S5.

CMM Pinyin	Scientific Name	Predicted ADR (MedDRA PT)	TCM System Mapping
HeShouWu	*Polygonum multiflorum* Thunb.	Hepatic function abnormal	Liver+Qi-Blood-Fluid
Hongchezhou-cao	*Trifolium pratense* L.	Drug-induced liver injury	Liver+Qi-Blood-Fluid
Hehuanpi	*Albizia julibrissin* Durazz.	Delirium	Heart
Yanhusuo	*Corydalis yanhusuo* W.T.Wang	Somnolence	Heart
Hongjingtian	*Rhodiola crenulata* (Hook.f. et Thomson) H.Ohba	Dizziness	Liver
Jixingzi	*Impatiens balsamina* L.	Dizziness	Liver
Daji	*Euphorbia pekinensis* Rupr.	Vomiting	Stomach
Longkui	*Solanum nigrum* L.	Nausea	Stomach
Suoyang	*Cynomorium songaricum* Rupr.	Vomiting	Stomach
Wuzhuyu	*Euodia rutaecarpa* (Juss.) Benth.	Hepatic function abnormal	Liver+Qi-Blood-Fluid
Tianhuafen	*Trichosanthes kirilowii* Maxim.	Vomiting	Stomach
Pipaye	*Eriobotrya japonica* (Thunb.) Lindl.	Urethral pain	Bladder
Danshen	*Salvia miltiorrhiza* Bunge	Abdominal pain	Spleen
Baiguo	*Ginkgo biloba* L.	Ear discomfort	Kidney
Zhugenqi	*Disporopsis fuscopicta* Hance	Vomiting	Stomach

Table S7. Validation of randomly sampled predictions from rank 101–200. We uniformly randomly sampled 15 predicted CMM–ADR pairs from the model's rank 101–200 list (lower-confidence stratum), after excluding labeled positive edges used for supervised evaluation, and applied the same two-channel validation protocol as in Table S5. Support is defined as database verification *or* literature/mechanistic support (logical OR).

CMM Pinyin	Scientific Name	Predicted ADR (MedDRA PT)	MSAT Score (%)	Database Verified	Mechanistic Support
Chaihu	*Bupleurum chinense* DC.	Hepatic function abnormal	92.850	Yes	Yes[a]
Pugongying	*Taraxacum mongolicum* Hand.-Mazz.	Constipation	91.950	Yes	No
Fuping	*Spirodela polyrhiza* (L.) Schleid.	Dry mouth	91.200	No	No
Sanleng	*Sparganium stoloniferum* Buch.-Ham.	Reproductive toxicity	90.550	Yes	Yes[b,c]
Gaoben	*Ligusticum sinense* Oliv.	Insomnia	89.880	No	No
Kunbu	*Laminaria japonica* Aresch.	Dry throat	88.940	No	No
Niuxi	*Achyranthes bidentata* Blume	Chest discomfort	88.120	No	No
Diyu	*Sanguisorba officinalis* L.	Tremor	87.450	No	No
Danzhuye	*Lophatherum gracile* Brongn.	Diarrhoea	86.660	No	No
Cheqiancao	*Plantago asiatica* L.	Tinnitus	85.800	No	No
Chuanxiong	*Ligusticum chuanxiong* Hort.	Diarrhoea	84.900	No	No
Baiziren	*Platycladus orientalis* (L.) Franco	Abdominal pain	84.150	No	No
Difuzi	*Kochia scoparia* (L.) Schrad.	Palpitations	83.500	No	No
Gaoliangjiang	*Alpinia officinarum* Hance	Rash	82.800	No	No
Baihe	*Lilium lancifolium* Thunb.	Vomiting	82.150	No	No

Mechanistic literature support: [a](Lee et al., 2011), [b](Sun et al., 2011), and [c](Jia et al., 2021).

Table S8. TCM functional system mapping for the randomly sampled rank 101–200 predictions. Functional system mapping for the 15 randomly sampled predictions in Table S7, using the same mapping framework as in the main text.

CMM Pinyin	Scientific Name	Predicted ADR (MedDRA PT)	TCM System Mapping
Chaihu	*Bupleurum chinense* DC.	Hepatic function abnormal	Liver+Qi-Blood-Fluid
Pugongying	*Taraxacum mongolicum* Hand.-Mazz.	Constipation	Large Intestine
Fuping	*Spirodela polyrhiza* (L.) Schleid.	Dry mouth	Stomach
Sanleng	*Sparganium stoloniferum* Buch.-Ham.	Reproductive toxicity	Chong and Ren Meridians
Gaoben	*Ligusticum sinense* Oliv.	Insomnia	Heart
Kunbu	*Laminaria japonica* Aresch.	Dry throat	Lung
Niuxi	*Achyranthes bidentata* Blume	Chest discomfort	Lung
Diyu	*Sanguisorba officinalis* L.	Tremor	Body Surface
Danzhuye	*Lophatherum gracile* Brongn.	Diarrhoea	Large Intestine
Cheqiancao	*Plantago asiatica* L.	Tinnitus	Kidney
Chuanxiong	*Ligusticum chuanxiong* Hort.	Diarrhoea	Large Intestine
Baiziren	*Platycladus orientalis* (L.) Franco	Abdominal pain	Spleen
Difuzi	*Kochia scoparia* (L.) Schrad.	Palpitations	Heart
Gaoliangjiang	*Alpinia officinarum* Hance	Rash	Liver+Qi-Blood-Fluid
Baihe	*Lilium lancifolium* Thunb.	Vomiting	Stomach

5 SUPPLEMENTARY FIGURES

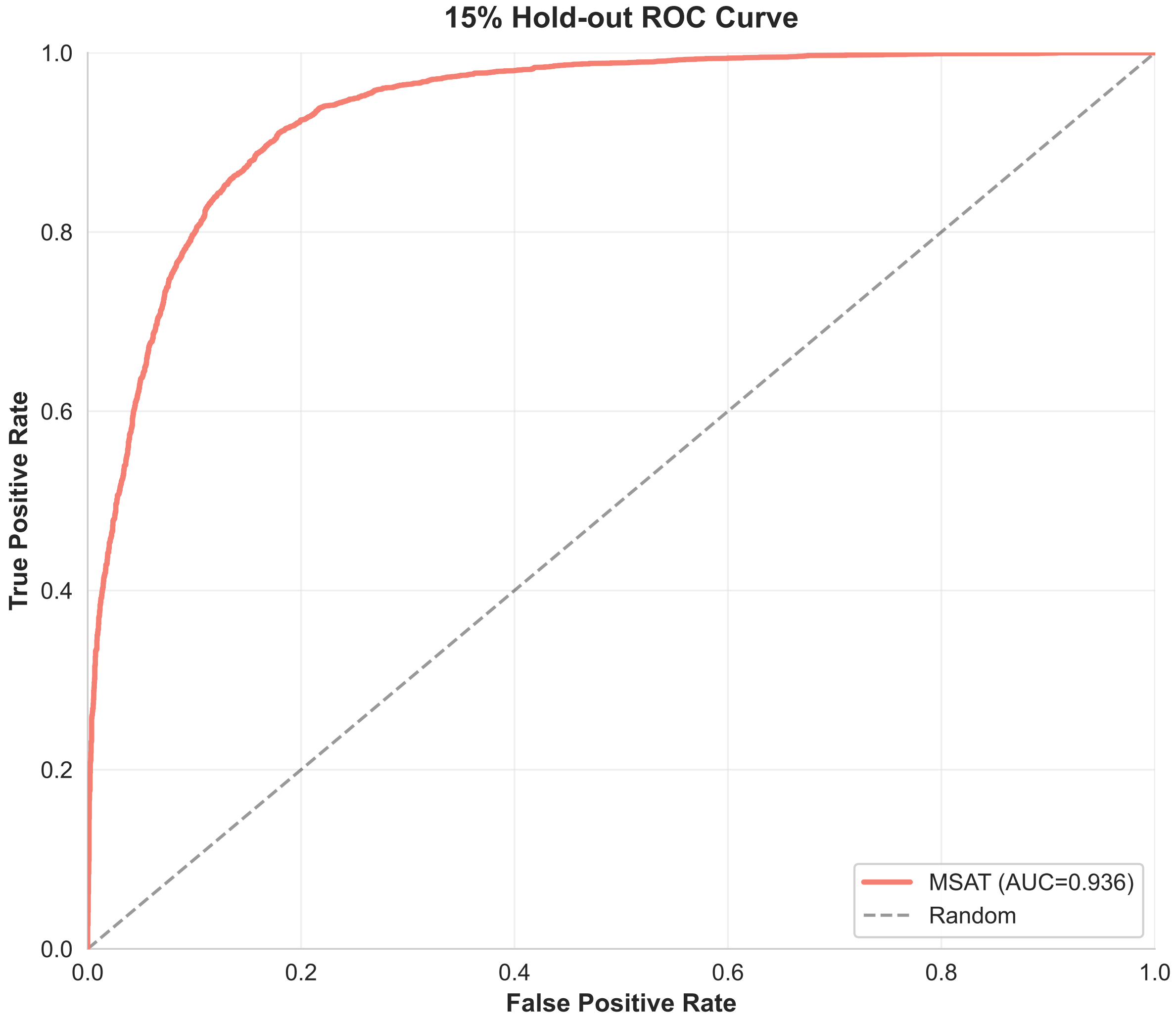

Figure S1. Supplementary Figure S1. 15% hold-out ROC curve.

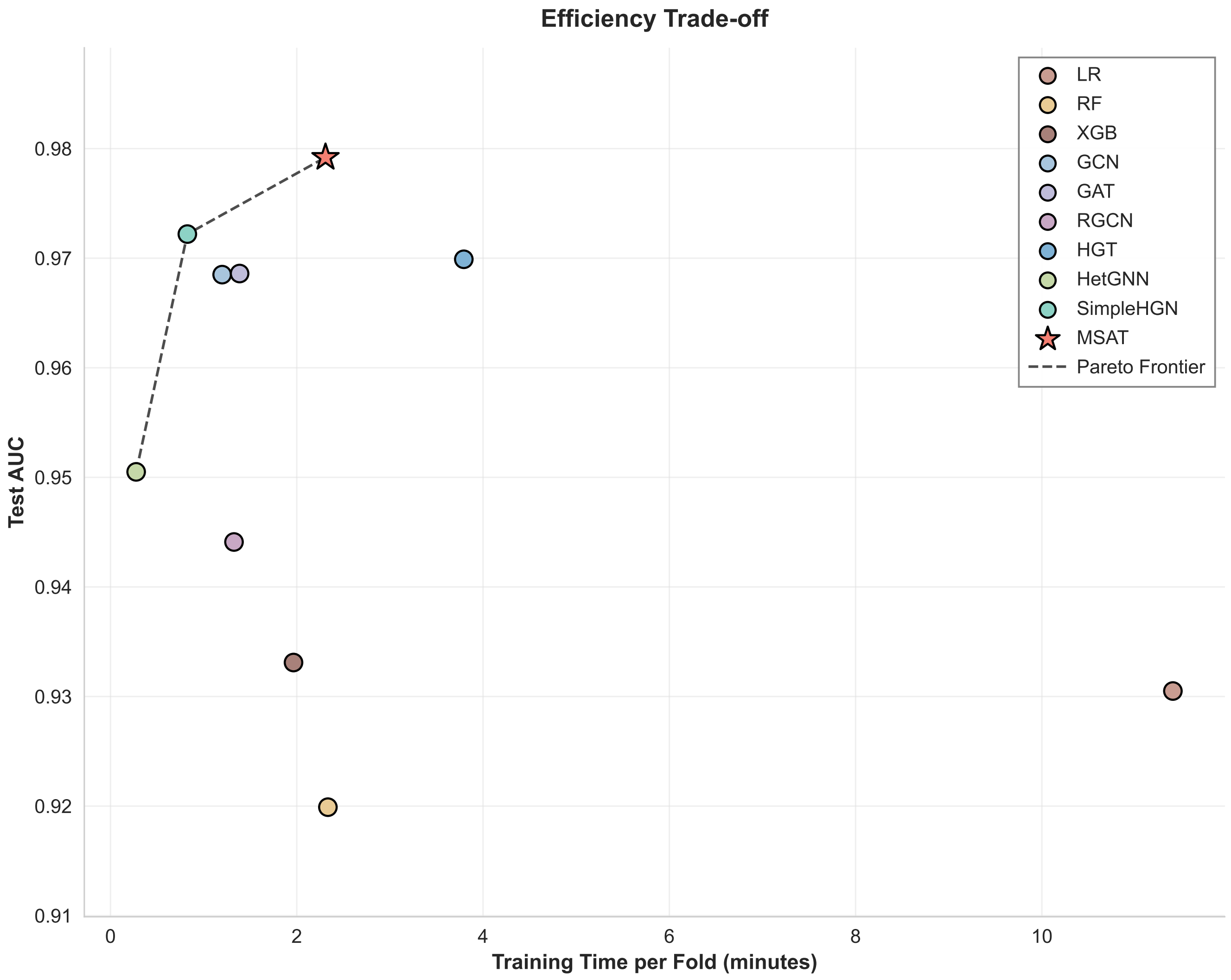

Figure S2. Supplementary Figure S2. Efficiency trade-off (test AUC versus training time per fold).

5.0.0.1 Operating points for pharmacovigilance workflows (reviewer request).

While AUPRC summarizes ranking performance under imbalance, practical deployment requires choosing a concrete decision threshold (an operating point) to trade off missed signals versus alert burden. Accordingly, we provide PR curves for the $1{:}10$ proxy setting and report two representative operating points: (i) a fixed $\tau{=}0.5$ threshold for comparability, and (ii) τ^* selected exclusively on the validation set (max-F1) to illustrate a data-driven thresholding strategy without test-set tuning.

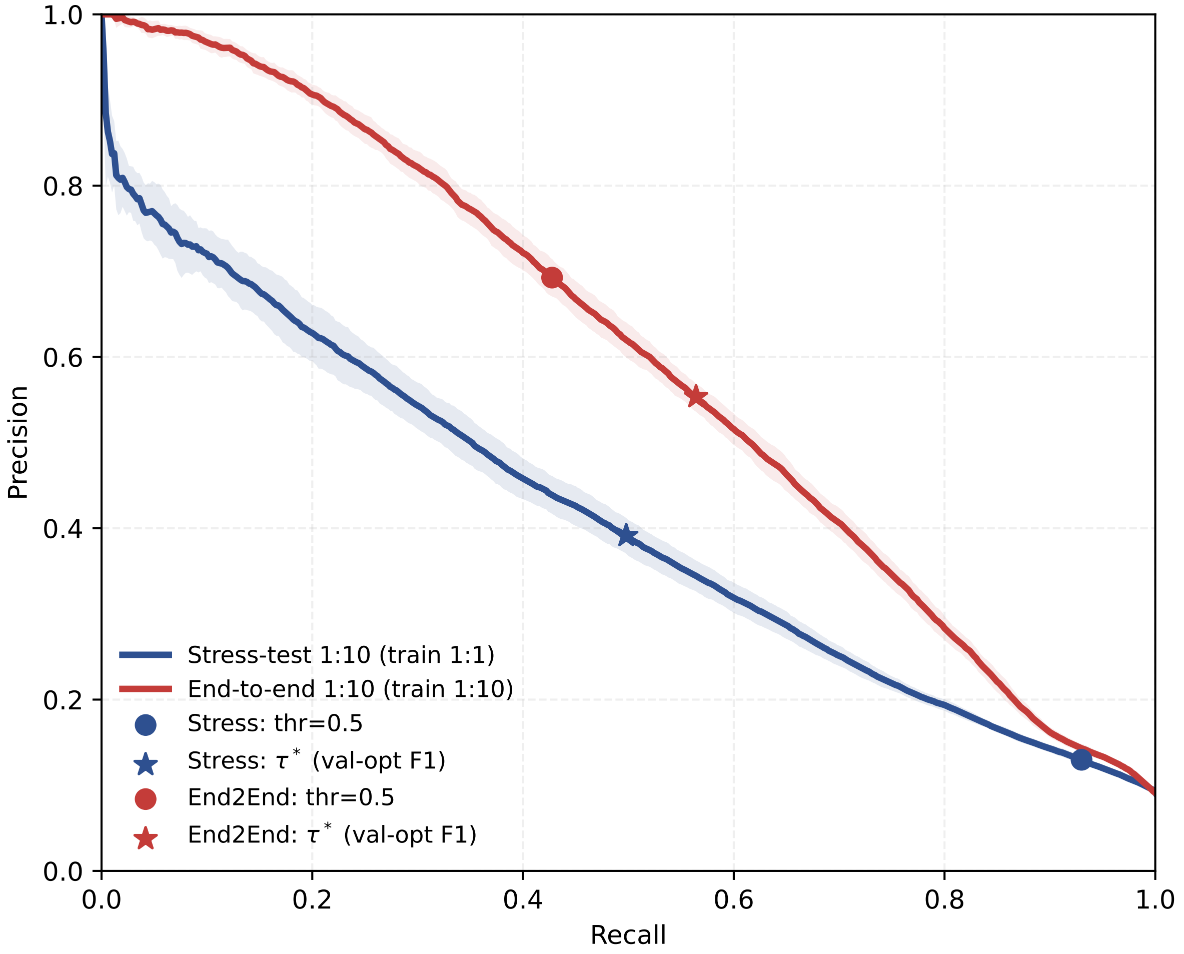

Figure S3. Supplementary Figure S3. Precision–recall curves under a $1{:}10$ class-imbalance proxy setting. We report PR curves (mean $\pm$ 1 std over 10 folds) under two complementary settings: (**i**) stress-test $1{:}10$ (train $1{:}1$, test $1{:}10$) and (**ii**) end-to-end $1{:}10$ (train/val/test $1{:}10$). Markers indicate two operating points: the default threshold ($\tau{=}0.5$) and a validation-selected threshold (τ^*) that maximizes the validation-set F1-score and is then applied unchanged to the matched test set.

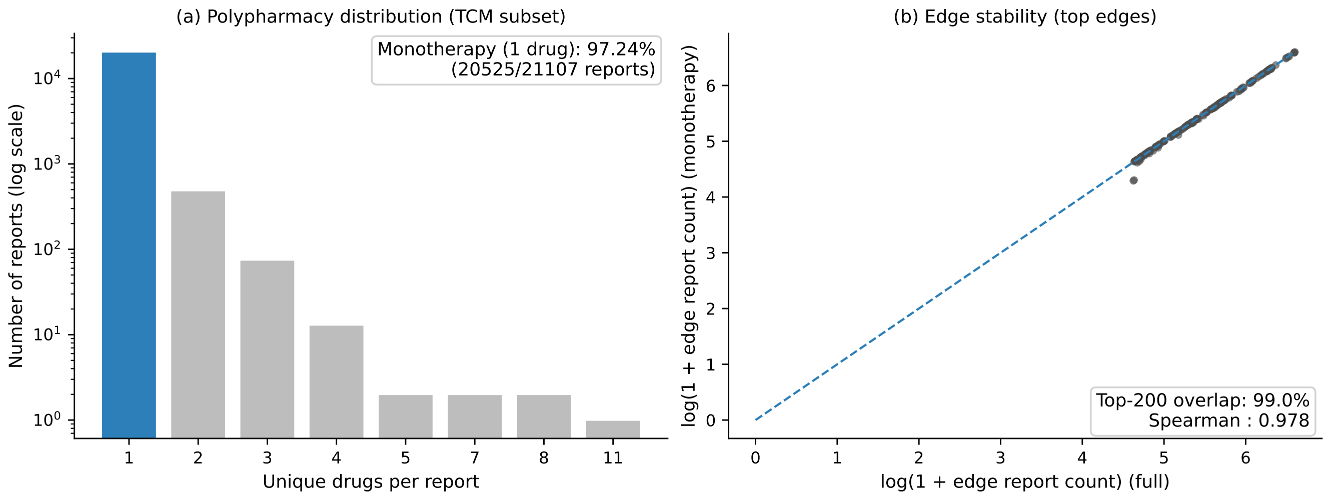

Figure S4. Supplementary Figure S4. FAERS preprocessing nuance: polypharmacy distribution and monotherapy-dominant sensitivity. (**a**) Distribution of the number of unique drugs per report in the exported TCM-containing FAERS table (log y-scale). (**b**) Robustness of CMM–ADR report-count edges when restricting to monotherapy-dominant reports (defined as reports with exactly one unique drug in the exported table), showing strong overlap and rank correlation with the full edge set (top edges, see annotation). This analysis is a robustness check for co-medication bias and does not establish causality.

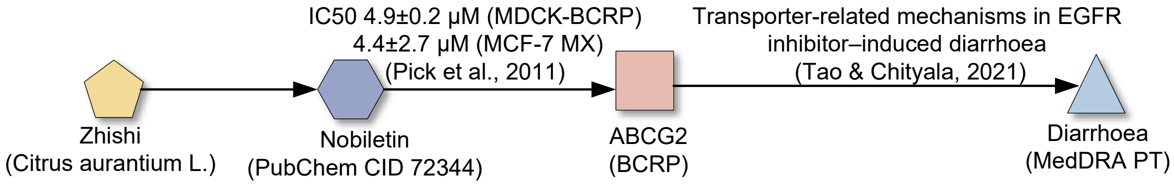

Figure S5. Supplementary Figure S5. Interpretability case study (path tracing, hypothesis-generating). Example post-hoc mechanistic path for a predicted CMM–ADR association: Zhǐ Shí (*Citrus aurantium* L.) $\rightarrow$ nobiletin (PubChem CID 72344) $\rightarrow$ ABCG2 (BCRP) $\rightarrow$ diarrhoea (MedDRA PT). Shown for interpretability and hypothesis generation, and it does not imply causality.

REFERENCES

Cai, Q., Wei, J., Zhao, W., Shi, S., Zhang, Y., Wei, R., et al. (2014). Toxicity of evodiae fructus on rat liver mitochondria: The role of oxidative stress and mitochondrial permeability transition. *Molecules* 19, 21168–21182. doi:10.3390/molecules191221168

Engelhardt, P. F. and Riedl, C. R. (2008). Effects of one-year treatment with isoflavone extract from red clover on prostate, liver function, sexual function, and quality of life in men with elevated psa levels and negative prostate biopsy findings. *Urology* 71, 185–190. doi:10.1016/j.urology.2007.08.068

Jia, J., Li, X., Ren, X., Liu, X., Wang, Y., Dong, Y., et al. (2021). Sparganii rhizoma: A review of traditional clinical application, processing, phytochemistry, pharmacology, and toxicity. *Journal of Ethnopharmacology* 268, 113571. doi:10.1016/j.jep.2020.113571

Lee, C.-H., Wang, J.-D., and Chen, P.-C. (2011). Risk of liver injury associated with chinese herbal products containing radix bupleuri in 639,779 patients with hepatitis b virus infection. *PLOS ONE* 6, e16064. doi:10.1371/journal.pone.0016064

Li, C., Rao, T., Chen, X., Zou, Z., Wei, A., Tang, J., et al. (2019). Hla-b*35:01 allele is a potential biomarker for predicting polygonum multiflorum-induced liver injury in humans. *Hepatology* 70, 346–357. doi:10.1002/hep.30660

Mao, J. J., Xie, S. X., Zee, J., Soeller, I., Li, Q. S., Rockwell, K., et al. (2015). Rhodiola rosea versus sertraline for major depressive disorder: A randomized placebo-controlled trial. *Phytomedicine* 22, 394–399. doi:10.1016/j.phymed.2015.01.010

Roelen, C., Mulder-Spijkerboer, H. N., Gee, E. S. I., Kolukirik, P., Biesta-Peters, E. G., and Royen, H. (2024). Public health risk due to contamination of Solanum nigrum in frozen green beans – collaboration effort between a poison centre, a hospital and health authorities. *Clinical Toxicology* 62, 126–128. doi:10.1080/15563650.2024.2320838

Sun, J., Wang, S., and Wei, Y.-H. (2011). Reproductive toxicity of rhizoma sparganii (sparganium stoloniferum buch.-ham.) in mice: mechanisms of anti-angiogenesis and anti-estrogen pharmacologic activities. *Journal of Ethnopharmacology* 137, 1498–1503. doi:10.1016/j.jep.2011.08.026

Wang, J. B. and Mantsch, J. R. (2012). l-tetrahydropalamatine: a potential new medication for the treatment of cocaine addiction. *Future Medicinal Chemistry* 4, 177–186. doi:10.4155/fmc.11.166

Zhang, J., Chen, X., Han, L., Ma, B., Tian, M., Bai, C., et al. (2024). Research progress in traditional applications, phytochemistry, pharmacology, and safety evaluation of cynomorium songaricum. *Molecules* 29, 941. doi:10.3390/molecules29050941
